# Multi‐Functional Actuators Made with Biomass‐Based Graphene‐Polymer Films for Intelligent Gesture Recognition and Multi‐Mode Self‐Powered Sensing

**DOI:** 10.1002/advs.202309846

**Published:** 2024-03-26

**Authors:** Mingcen Weng, Jiahao Zhou, Peidi Zhou, Ruzhi Shang, Minghua You, Guozhen Shen, Huamin Chen

**Affiliations:** ^1^ School of Materials Science and Engineering Fujian Provincial Key Laboratory of Advanced Materials Processing and Application Key Laboratory of Polymer Materials and Products of Universities in Fujian Fujian University of Technology Fuzhou Fujian 350118 China; ^2^ Institute of Smart Marine and Engineering Fujian University of Technology Fuzhou Fujian 350118 China; ^3^ Fujian Key Laboratory of Functional Marine Sensing Materials College of Materials and Chemical Engineering Minjiang University Fuzhou 350108 China; ^4^ College of Mechanical and Electrical Engineering Fujian Agriculture and Forestry University Fuzhou 350108 China; ^5^ School of Integrated Circuits and Electronics Beijing Institute of Technology Beijing 100081 China

**Keywords:** flexible electronics, machine learning, multi‐functional actuator, multi‐mode sensing, self‐powered device

## Abstract

Multi‐functional actuation systems involve the mechanical integration of multiple actuation and sensor devices with external energy sources. The intricate combination makes it difficult to meet the requirements of lightweight. Hence, polypyrrole@graphene‐bacterial cellulose (PPy@G‐BC) films are proposed to construct multi‐responsive and bilayer actuators integrated with multi‐mode self‐powered sensing function. The PPy@G‐BC film not only exhibits good photo‐thermoelectric (PTE) properties but also possesses good hydrophilicity and high Young's modulus. Thus, the PPy@G‐BC films are used as active layers in multi‐responsive bilayer actuators integrated with self‐powered sensing functions. Here, two types of multi‐functional actuators integrated with self‐powered sensing functions is designed. One is a light‐driven actuator that realizes the self‐powered temperature sensing function through the PTE effect. Assisted by a machine learning algorithm, the self‐powered bionic hand can realize intelligent gesture recognition with an accuracy rate of 96.8%. The other is humidity‐driven actuators integrated a zinc‐air battery, which can realize self‐powered humidity sensing. Based on the above advantages, these two multi‐functional actuators are ingeniously integrated into a single device, which can simultaneously perform self‐powered temperature/humidity sensing while grasping objects. The highly integrated design enables the efficient utilization of environmental energy sources and complementary synergistic monitoring of multiple physical properties without increasing system complexity.

## Graphical abstract

Polypyrrole@graphene‐bacterial cellulose films are proposed to construct multi‐responsive actuators integrated with self‐powered and multi‐mode sensing function. Two types of multi‐functional actuators are ingeniously integrated into a single device, which can simultaneously perform self‐powered temperature/humidity sensing while grasping objects. The highly integrated design enables the efficient utilization of environmental energy sources and complementary synergistic monitoring of multiple physical properties.

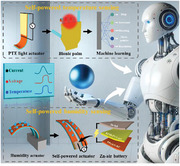

## 1 Introduction

The continuous development and innovation of artificial intelligence technology has promoted the wide application of smart actuators with excellent actuation performance in fields such as flexible electronics,^[^ ^1^, ^2^, ^3^ ^]^ soft robots,^[^ ^4^, ^5^, ^6^ ^]^ and biomimetic applications.^[^ ^7^, ^8^, ^9^ ^]^ Due to the increasingly complex tasks required to be performed by the new generation of intelligent robots, the integration of various practical functions on traditional actuators is an inevitable path for the future development of smart actuators. Some researchers have attempted to integrate sensing functions on flexible actuators to monitor the external stimuli received in real time by collecting feedback electrical signals.^[^ ^10^, ^11^, ^12^, ^13^ ^]^ However, the portability and monitoring duration of these actuation systems integrated with sensing functions inevitably deteriorates because of the energy consumption associated with complex external power supplies and connecting wires.

As a novel micro‐power generator, the triboelectric nanogenerator (TENG) can convert the charge in the tribolayer into an electrical signal through a contact‐separation mode.^[^ ^14^, ^15^ ^]^ TENG's in‐depth research provides a new solution to construct all‐in‐one actuators with both actuation and sensing functions. As a result, there have been several studies reported on integrating TENG inside or outside of photo‐thermal actuators, pneumatic actuators, and vapor actuators for the preparation of mechanical grippers with self‐powered sensing functions.^[^ ^16^, ^17^, ^18^ ^]^ For instance, Wang et al. designed a polyethylene terephthalate‐carbon black ink‐polydimethylsiloxane actuator with excellent robustness and contact feedback that can simulate a frog's tongue and mechanical gripper to bend and output triboelectric voltage under light‐induced.^[^ ^16^ ^]^ Chen et al. prepared a sensorized pneumatic gripper using silicone rubber and conductive sponge material, which can recognize the size and weight of the gripped object through self‐powered voltage.^[^ ^17^ ^]^ However, the application range of TENG‐based self‐powered actuator devices is restricted due to their high preparation costs and the necessity for dynamic contact to generate electrical signals. In contrast, the photo‐thermoelectric generator (PTEG) is a passive energy conversion device working based on the Seebeck effect, which can convert thermal energy from the environment into electrical energy contactless under the condition of temperature difference. Several researchers have combined PTEG in situ on photo‐thermal actuators to prepare flexible photo‐thermal actuators with self‐powered sensing functions.^[^ ^19^, ^20^, ^21^ ^]^ For example, Chen et al. prepared a graphite/paper/thermochromic dye photo‐thermal material by the pencil‐on‐paper method and applied it to a light‐driven actuator with an integrated self‐powered/visual dual‐mode sensing function and rewritable display function.^[^ ^19^ ^]^ Weng et al. proposed a Ti_3_C_2_T_X_‐based composite modified by bamboo nanofibers, based on which a light/electro‐driven actuator was developed to accomplish Marangoni floating and self‐powered temperature sensing.^[^ ^21^ ^]^ Benefiting from the wireless actuation characteristics of the light source, the entire energy conversion process can be easily controlled without external force. In addition, PTEGs share similar actuation and energy utilization modes with light‐driven actuators based on the deformation of the asymmetric photo‐thermal expansion mechanism.^[^ ^22^ ^]^ Nevertheless, none of the above studies were able to realize both multi‐responsive actuation and multi‐mode self‐powered sensing functions in a single device. Integrating multi‐responsive actuation and self‐powered sensing in a single device not only allows the device to more fully utilize the energy resources in the environment (e.g., light energy, moisture, etc.) but also enables complementary monitoring of multiple physical properties of itself (e.g., temperature, water content, etc.) without increasing the complexity of the device. Recently, the flexible zinc‐air battery consisting of flexible electrodes and solid electrolytes has gradually become a reliable and promising power device due to its high energy density, environmental friendliness, and inexpensive cost.^[^ ^23^, ^24^ ^]^ We notice that some reported humidity bilayer actuators possess humidity‐sensitive layers with high specific surface area and excellent electrical conductivity, which are well suited as electrodes for zinc‐air batteries.^[^ ^22^, ^25^, ^26^ ^]^ Also, in situ integration of zinc‐air batteries in actuators has not been reported, as far as we know. Therefore, a novel actuator with a self‐powered sensing function could potentially be created by integrating the zinc‐air battery in situ on the bilayer actuator.

**Scheme 1:**
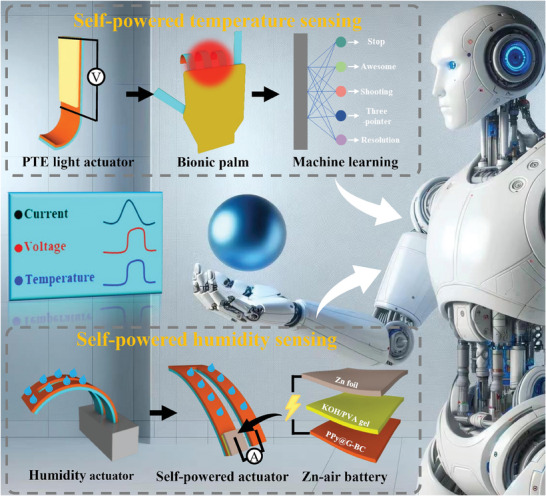
Intelligent gripper based on the multi‐responsive PPy@G‐BC/BOPP actuators integrated with self‐powered and multi‐mode sensing functions. Background image is designed by Midjourney.

Herein, the free‐standing polypyrrole@graphene‐bacterial cellulose (PPy@G‐BC) films were prepared by using a low‐cost method combining vacuum filtration and in‐situ polymerization. Through extensive hydrogen bonding, the G‐BC films with a multilayer network structure were constructed by stacking hydrophilic BC nanofibers and graphene nanosheets with excellent photo‐thermal conversion capabilities. In addition, a large number of conductive polypyrrole nanoparticles were uniformly filled into these network pores of G‐BC film through in situ polymerization. The prepared PPy@G‐BC film was laminated with a biaxially oriented polypropylene (BOPP) film via acrylic ester to construct a light/humidity‐driven bilayer actuator. Hydrophobic BOPP film possesses a larger coefficient of thermal expansion (CTE) than the PPy@G‐BC film.^[^ ^27^ ^]^ Thanks to the excellent photo‐thermal conversion and thermoelectric properties, the PPy@G‐BC layer spontaneously converts the temperature difference of the actuator into a thermoelectric signal through the Seebeck effect during the asymmetric thermal expansion with the BOPP layer. The thermoelectric signal generated by the PPy@G‐BC/BOPP actuator can realize self‐powered temperature sensing. With the assistance of a machine learning algorithm, a self‐powered bionic hand designed based on PPy@G‐BC/BOPP actuators can accurately differentiate the spontaneously generated thermoelectric signals to realize gesture recognition. Furthermore, the highly conductive PPy@G‐BC layer also provides a good air electrode platform for the in situ integration of flexible zinc‐air batteries. The PPy@G‐BC/BOPP actuator with an in‐situ integrated zinc‐air battery can reflect the environmental humidity and its water content change in real‐time. The current signal change of the PPy@G‐BC/BOPP actuator results from the humidity resistance effect of the PPy@G‐BC film. Notably, the self‐powered electrical signals of these two PPy@G‐BC/BOPP actuators are in good consistency with their deformation state and physical properties. Finally, the two aforementioned multi‐functional actuators were highly integrated into a single intelligent gripper device, realizing multi‐responsive actuation and self‐powered multi‐mode sensing function, as shown in **Scheme** **1**. The intelligent gripper can not only move objects steadily but also monitor the physical properties (temperature, humidity, and water content) and deformation state of the actuators in a complementary and synergistic way. The innovative design of integrating multi‐responsive actuation and multi‐mode self‐powered sensing in a single flexible electronic device is expected to assist intelligent soft robots in complex and changing environments to better perceive and perform their tasks.

## 2 Result and Discussion

### 2.1 Fabrication and Characterization of PPy@G‐BC Film

**Figure 1:**
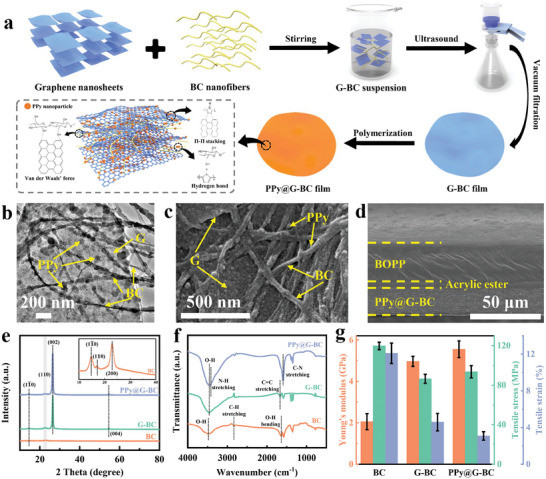
Fabrication and characterization of the PPy@G‐BC film. a) Schematic diagram of the fabrication process of the PPy@G‐BC film. b) TEM image of the PPy@G‐BC film. c) SEM image of the surface of PPy@G‐BC film. d) Cross‐section SEM image of the PPy@G‐BC/BOPP actuator. e) The XRD spectra of BC film, G‐BC film, and PPy@G‐BC film. f) The FTIR spectra of BC film, G‐BC film, and PPy@G‐BC film. g) Mechanical properties of BC film, G‐BC film, and PPy@G‐BC film (sample size = 5).

Graphene is an ultrathin 2D layered material that is composed of carbon atoms in sp^2^ hybridization (Figure S1, Supporting Information). Thanks to its excellent properties in photo‐thermal conversion, thermoelectricity, and electricity, graphene has become one of the most promising materials in the fields of actuators, thermoelectric generators, and nanogenerators.^[^ ^28^, ^29^, ^30^ ^]^ However, pure graphene film generally lacks the abilities of free‐standing and hydrophilicity, which makes it difficult to meet the requirements of multi‐functional and multi‐responsive actuators.^[^ ^20^ ^]^ The preparation process of PPy@G‐BC film is schematically shown in **Figure** **1**a. As a natural biomass material, there are a large number of oxygen‐containing functional groups on the surface of the BC nanofibers that can generate strong hydrogen bonding with water molecules.^[^ ^26^ ^]^ What's more, as shown in Figure S2 (Supporting Information), the tightly cross‐linking porous network structure also endows BC nanofibers with satisfactory strength and toughness.^[^ ^31^ ^]^ Therefore, BC nanofibers with excellent hydrophilic and mechanical properties were selected as the substrate. Uniform G‐BC dispersion was obtained by dispersing BC nanofibers and graphene nanosheets in deionized water by magnetic stirring and ultrasonic dispersion methods. Then, assisted by the vacuum filtration method, graphene nanosheets and BC nanofibers were alternately stacked together through extensive hydrogen bonding interactions and van der Waals’ forces, thereby forming a free‐standing film. The prepared G‐BC films exhibited a gray color (Figure S3a, Supporting Information). The TEM images depicted in Figure S4 (Supporting Information) illustrate G‐BC films at various magnifications, revealing the tight combination of graphene nanosheets with BC nanofibers. Besides, from the SEM images of the surface and cross‐section of the G‐BC film shown in Figure S5a–d (Supporting Information), it can be seen that the BC nanofibers tightly wrapped the graphene nanosheets, constructing a stable multilayer network structure through surface interaction forces. Although the mechanical properties and hydrophilicity of G‐BC film gain improvement compared to pure graphene film, the insulating properties of BC nanofibers will inevitably deteriorate the electrical conductivity of graphene. To address this issue, an in‐situ polymerization method was employed to grow highly conductive PPy nanoparticles on the G‐BC film. As shown in Figure 1b,c, the Py monomers grow in situ into pearl‐like PPy nanoparticles under the effect of oxidizing agents, which then uniformly attach to the surfaces and gaps of the G‐BC porous network structure by π‐π stacking and hydrogen bonding interactions. Compared to the G‐BC film, the resulting PPy@G‐BC film exhibits a darker black color (Figure S3b, Supporting Information), indicating successful polymerization. Notably, the prepared PPy@G‐BC film shows excellent flexibility (Figure S6a, Supporting Information). More experimental details are described in the Experimental Section. Since the BOPP film possesses excellent mechanical properties, the PPy@G‐BC/BOPP actuator with a bilayer structure also exhibits exceptional flexibility (Figure S6b, Supporting Information). The SEM image of the cross‐section of the PPy@G‐BC/BOPP actuator is shown in Figure 1d. It can be seen that the BOPP film is bonded to the PPy@G‐BC film by the adhesion of acrylic ester. In order to investigate the bonding force between the bilayers of the PPy@G‐BC/BOPP actuator, a peeling test was performed, and the test results are shown in Figure S7 (Supporting Information). The peeling force between the BOPP layer and the PPy@G‐BC layer was measured to be up to 10.12 MPa, indicating that there is an excellent bonding force between these two layers. The pressure‐sensitive acrylic ester (≈8 µm in thickness) is able to bond the PPy@G‐BC layer to the BOPP layer by molecular forces under pressure, thus providing a strong interaction force. As a result, the heat converted by the PPy@G‐BC layer can be quickly transferred to the BOPP layer. The thickness of the PPy@G‐BC film is ≈26 µm, and the thickness of the PPy@G‐BC/BOPP actuator is ≈64 µm.

XRD was first used to analyze the crystal structures of BC film, G‐BC film, and PPy@G‐BC film, as shown in Figure 1e. The prepared BC film exhibits three typical diffraction peaks at 14.8°, 17.04°, and 22.9°, which correspond to the (1$\bar{1}$0), (110), and (200) cellulose I crystal planes, respectively.^[^ ^32^ ^]^ The G‐BC film exhibits a strong diffraction peak at 26.4° and a weak diffraction peak at 54.5°, corresponding to the (002) and (004) crystal planes of graphene.^[^ ^33^ ^]^ The above result demonstrates that graphene maintains a good lattice structure and lamellar spatial arrangement after the introduction of BC nanofibers. After the polymerization of PPy, no obvious new diffraction peaks were observed in the PPy@G‐BC film. However, the characteristic broad peak at 26.4° is significantly enhanced, which is due to the superposition of the diffraction peaks of the introduced PPy with graphene.^[^ ^34^, ^35^ ^]^ The FTIR spectra of BC film, G‐BC film, and PPy@G‐BC film are shown in Figure 1f. Three typical characteristic peaks at 3478, 2836, and 1641 cm^−1^ appeared in the BC film, which corresponded to the stretching vibration of hydrogen bonding induced by the O‐H group, the asymmetric stretching vibration of C‐H in the parabolic ring, and the O‐H bending vibration, respectively.^[^ ^36^ ^]^ After the introduction of graphene nanosheets, a diffraction peak appears at 1673 cm^−1^ in the G‐BC film, corresponding to the C = C stretching vibration on the benzene‐like ring structure in graphene.^[^ ^37^ ^]^ In addition, the diffraction peak corresponding to the O‐H stretching vibration in the G‐BC film is red‐shifted from 3478 cm^−1^ to a shorter wavenumber of 3451 cm^−1^, compared to the BC film. This is because the strong hydrogen bonding interaction between graphene nanosheets and BC nanofibers leads to the movement of the stretching vibration bands of O‐H, which are susceptible to hydrogen bonding interaction.^[^ ^38^, ^39^ ^]^ Typical characteristic peaks of PPy can be observed at 3420 cm^−1^ (N‐H stretching vibration) and 1588 cm^−1^ (asymmetric C‐N ring‐stretching vibration) for the PPy@G‐BC film, which indicates that PPy nanoparticles successfully grew on the G‐BC film.^[^ ^40^ ^]^ Then, the mechanical properties of BC film, G‐BC film, and PPy@G‐BC film were also characterized, as shown in Figure 1g and Figure S8 (Supporting Information). The graphene doping can lead to a Young's modulus of 4.96 GPa for the G‐BC film, which is 2.4 times higher than that of the pure BC film (2.05 GPa). The phenomenon may be attributed to the fact that graphene nanosheets can enhance the connectivity of the BC network structure and hinder the twisting and buckling of BC nanofibers. After polymerization of PPy nanoparticles, the mechanical properties of PPy@G‐BC film were also enhanced compared to G‐BC film, with its Young's modulus and tensile stress enhanced by ≈1.12 times (4.96–5.55 GPa) and 1.08 times (86.41–93.53 MPa), respectively. Compared to the similar flexible materials in the field of multifunctional actuators, the mechanical properties (especially Young's modulus and tensile stress) of the PPy@G‐BC film exhibit a high level, as shown in Table S1 (Supporting Information). This is because PPy nanoparticles can form tight adsorptions on the surface and in the gaps between BC nanofibers and graphene nanosheets through π–π stacking and hydrogen bonding interactions.^[^ ^35^, ^41^ ^]^ As a result, PPy nanoparticles act as mechanically enhanced fillers to strengthen the mutual attraction between the nanomaterials and fill the structural gaps as well, which endows the PPy@G‐BC film with a compact internal structure. Furthermore, to evaluate the mechanical stability of the PPy@G‐BC film, it was fixed on a displacement platform by BOPP film for 300 bending cycle tests (Figure S9, Supporting Information). It is found that the internal resistance of the PPy@G‐BC film only increased from 30.29 to 31.83 Ω after 300 cycles without significant surface damage. The test result demonstrates the excellent overall mechanical stability of the PPy@G‐BC film. Benefiting from the above properties, PPy@G‐BC film is expected to serve as a multi‐functional layer for broad applications in the field of multi‐functional actuators.

### 2.2 Thermoelectric Properties of PPy@G‐BC Film

**Figure 2:**
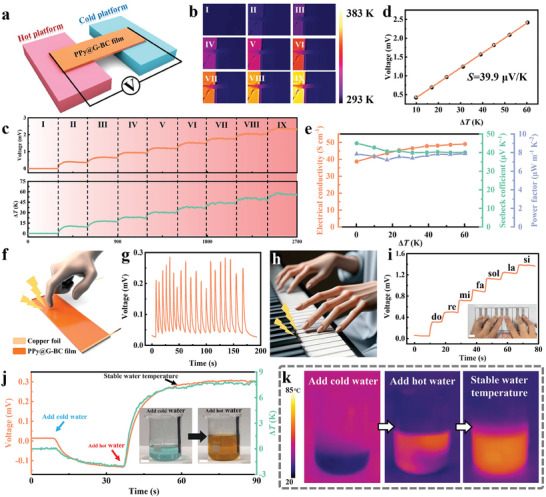
TE property of PPy@G‐BC film. a) Schematic diagram of the TE test. b) Infrared thermal images of the PPy@G‐BC film under different temperature differences. c) Δ*T* and *V*_oc_ of the PPy@G‐BC film heated by a hot plate. d) The *V*_oc_ of the PPy@G‐BC film as a function of Δ*T*. e) Electrical and thermoelectric properties of the PPy@G‐BC film under different temperature differences. f) Schematic diagram of the PPy@G‐BC film used as a thermoelectrical touch sensor. g) The *V*_oc_ of the PPy@G‐BC touch sensor under multiple touches. h) Schematic diagram of the “self‐powered piano” formed by multiple PPy@G‐BC films. Image is designed by Midjourney. i) The *V*_oc_ of the “self‐powered piano”, and the inset shows the corresponding optical photo. Scale bar: 2 cm. j) The *V*_oc_ and Δ*T* of the PPy@G‐BC film as a water temperature sensor, and the inset showing the corresponding optical photos. Scale bar: 2 cm. k) Infrared thermal images of the beaker integrated with the PPy@G‐BC water temperature sensor.

To investigate the thermoelectric properties of PPy@G‐BC film, the heat platform and the cold platform were used as heat source and cold source, respectively (**Figure** **2**a). In this way, a spatial temperature difference can be created. It is worth noting that high‐temperature‐resistant polyimide tapes were used to secure the electrodes at both ends of the PPy@G‐BC film. It can prevent the bending deformation of PPy@G‐BC film due to high temperature from affecting the accuracy of the thermoelectric property test. The left (1 cm) and right (1 cm) ends of the PPy@G‐BC film, fixed on the heat and cold platforms, served as the hot and cold ends of the thermoelectric generator. Meanwhile, the middle part (1 cm) was suspended. Specific experimental details and test structures are given in the Experimental Section and Figure S10 (Supporting Information). When the left heat platform starts to heat up, the hot and cold ends of the PPy@G‐BC film with almost the same initial temperature start to generate a temperature difference Δ*T*. According to the Seebeck effect, the current carriers (electric holes) in the PPy@G‐BC film will move from the hot end to the cold end, thus generating an output voltage. The Seebeck coefficient is one of the most important indicators used to characterize the thermoelectric conversion efficiency of a material. As shown in Figure 2b, nine different temperature segments were set on the heating platform to create the temperature gradient, which is necessary for the Seebeck coefficient test. Each temperature segment lasted for ≈300 s. The curve of the open‐circuit voltage *V*_oc_ of PPy@G‐BC film with temperature difference Δ*T* is shown in Figure 2c. Apparently, *V*_oc_ follows the same trend as the temperature gradient, showing a good linear correlation. *V*_oc_ can reach 2.417 mV at a Δ*T* of 60.3 K. The association between *V*_oc_ and Δ*T* is illustrated in Figure 2d. According to the formula of the Seebeck coefficient: *S* = *V*_oc_ /Δ*T* (*S* is the Seebeck coefficient of the thermoelectric generator), the Seebeck coefficient of PPy@G‐BC is calculated as 39.9 µV K^−1^. Under the same temperature gradient (upper panel of Figure 2c), the measured short‐circuit current *I*_sc_ (Figure S11, Supporting Information) of the PPy@G‐BC film also exhibited good consistency with the trend of Δ*T*. The above results reveal the excellent thermoelectric properties of PPy@G‐BC film, indicating it as an ideal flexible thermoelectric material. Apart from high Seebeck coefficients, ideal thermoelectric materials also require excellent electrical properties, which can directly affect the thermoelectric performance.^[^ ^42^ ^]^ Therefore, the electrical properties of the PPy@G‐BC film were characterized under different temperature differences, as shown in Figure 2e. The test results indicate that the PPy@G‐BC film possesses excellent electrical conductivity, which increases with the temperature gradient from 37.73 to 49.07 S cm^−1^. In addition, the highest power factor of the PPy@G‐BC film can reach 7.88 µW m^−1^ K^−2^, indicating its good energy utilization when used as a thermoelectric generator. Then, to investigate whether the in‐situ polymerization of PPy would affect the thermoelectric properties, the thermoelectric and electrical properties of G‐BC films were also tested under different temperature differences (Figures S12 and S13, Supporting Information). The Seebeck coefficient, electrical conductivity, and power factor of the G‐BC film were tested to be 19.4 µV K^−1^, 5.85 S cm^−1^, and 0.23 µW m^−1^ K^−2^, which were much lower than the corresponding properties of the PPy@G‐BC film. It is obvious that both the thermoelectric and electrical properties of PPy@G‐BC films are substantially improved after the introduction of highly conductive PPy nanoparticles. The enhancement of the thermoelectric properties of PPy@G‐BC film is mainly attributed to three factors. First, the introduction of highly conductive PPy nanoparticles can improve the electrical conductivity of the PPy@G‐BC film, thus accelerating the carrier migration rate. Second, PPy nanoparticles can effectively reduce the thermal conductivity of the PPy@G‐BC film, as shown in Figure S14a (Supporting Information). According to Note S3 (Supporting Information), the reduction of thermal conductivity can enhance the *ZT* value of the PPy@G‐BC film, which is a quality factor that measures the comprehensive thermoelectric properties of a material. Third, as a good thermoelectric material (≈14.5 µV K^−1^), the extensive interface combination of PPy nanoparticles significantly enhanced the Seebeck coefficient of the film from 19.4 µV K^−1^ to 39.9 µV K^−1^.^[^ ^43^ ^]^ Consequently, the *ZT* values of the PPy@G‐BC film obtained ≈60 times enhancement compared to the G‐BC film, indicating that PPy nanoparticles can substantially improve the comprehensive thermoelectric properties of the film (Figure S14b, Supporting Information). Furthermore, as can be seen from Table S2 (Supporting Information), the comprehensive thermoelectric properties of the PPy@G‐BC films are at an intermediate level among the same type of thermoelectric materials, which is sufficient for self‐powered thermoelectric sensing applications.

To evaluate the self‐powered performance and temperature sensitivity of the PPy@G‐BC film in practical applications, the film was employed as a thermoelectric touch sensor to detect finger touch movements directly (Figure 2f). As shown in Figure 2g, the PPy@G‐BC touch sensor can respond quickly to successive finger touch actions by generating thermoelectric voltage spontaneously. The result demonstrates the excellent thermoelectric properties and temperature sensitivity of the PPy@G‐BC touch sensor, which can sensitively detect temperature differences and quickly recover to the initial state. Next, an interesting “self‐powered piano” formed by connecting multiple PPy@G‐BC films in series was developed, as shown in Figure 2h. Due to the exceptional temperature sensitivity of each PPy@G‐BC film, the “self‐powered piano” can spontaneously generate stepped voltages in various touch modes. This allows for the simulation of musical scales, as shown in Figure 2i. Finally, the PPy@G‐BC film was attached directly to a beaker for utilization as a water temperature sensor. As can be seen in Figure 2j,k, after adding cold and hot water to the beaker successively, the PPy@G‐BC water temperature sensor can rapidly output the corresponding voltage signal with the temperature difference. Notably, such a water temperature sensing strategy can not only detect the time of adding cold or hot water but also reflect the stable water temperature. The result indicates that PPy@G‐BC film can be used as a practical water temperature sensor with high sensitivity. In addition, two tests were performed to evaluate the stability and durability of the PPy@G‐BC film for thermoelectric applications (Figure S15, Supporting Information). As can be seen from the results, the PPy@G‐BC film not only exhibits good stability in the long‐term thermoelectric performance test of 3600 s, but also stably outputs thermoelectric signals in the repeated thermostatic touch test. Therefore, the PPy@G‐BC film was demonstrated to be a flexible thermoelectric material with excellent temperature sensitivity, stability, and durability in thermoelectric applications.

### 2.3 Light‐Driven PPy@G‐BC/BOPP Actuator Integrated with Self‐Powered Temperature Sensing Function

**Figure 3:**
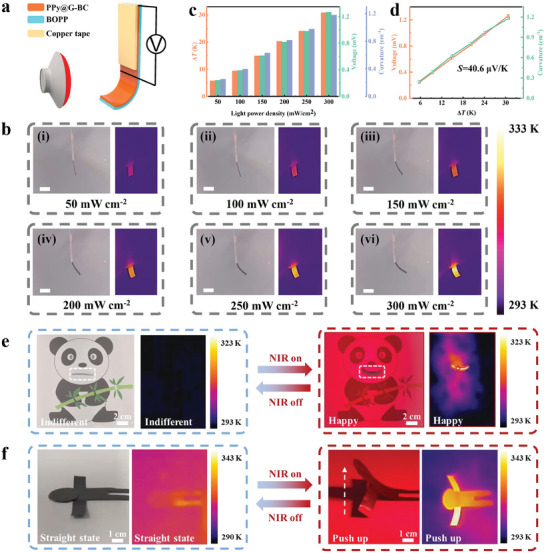
Light‐driven actuation and self‐powered sensing performances of the PPy@G‐BC/BOPP actuator. a) Schematic diagram of the light‐driven actuation test. b) Infrared thermal images of the PPy@G‐BC/BOPP actuator under different light power densities. Scale bar: 1 cm. c) Δ*T*, *V*_oc_, and curvature of the PPy@G‐BC/BOPP actuator under different light power densities. d) *V*_oc_ and curvature of the PPy@G‐BC/BOPP actuator as a function of Δ*T* when irradiated by NIR light. The curvatures were calculated according to Figure S18 and Note S1 (Supporting Information). e) Optical photos and corresponding infrared thermal images of the “Panda” robot from an indifferent state to a happy state. f) Optical photo and corresponding infrared thermal image of the “athlete” robot from a straight state to a push‐up state.

In recent years, PPy and graphene have been widely applied to PTEGs and light‐driven actuators due to their excellent photo‐thermal conversion and thermoelectric properties.^[^ ^44^, ^45^, ^46^, ^47^ ^]^ To overcome the limitations of the photo‐thermal conversion properties of a single material, graphene and PPy were combined to achieve more efficient photo‐thermal conversion. As shown in Figure S16 (Supporting Information), the PTE performance of the PPy@G‐BC/BOPP actuator was tested using copper tape as a photomask. The actuator was secured by a BOPP film on a U‐shaped glass frame. Near‐infrared (NIR) light was used as a light source. Specific experimental details and test structures are shown in the Experimental Section and Figure S16 (Supporting Information). The PPy@G‐BC/BOPP actuator was irradiated under NIR light with different light power densities, and the corresponding infrared thermal images are shown in Figure S17a (Supporting Information). Based on the PTE effect, the PPy@G‐BC/BOPP actuator can convert the temperature difference into output voltage, as shown in Figure S17b (Supporting Information). With the increase in light power density, the maximum Δ*T* and maximum *V*_oc_ show the same increasing trend. The calculated Seebeck coefficient of the PPy@G‐BC/BOPP actuator is 42.8 µV K^−1^, which is similar to the result obtained with the hot platform (Figure S17c, Supporting Information). In addition, the PPy@G‐BC/BOPP actuator maintained stable photo‐thermal conversion capability and self‐powered output capability under 300 cycle tests, as shown in Figure S17d (Supporting Information). The above results indicate that the PPy@G‐BC/BOPP actuator possesses excellent PTE properties. It is known that flexible light‐driven bilayer actuators are novel devices that can remotely realize mechanical actuation based on the photo‐thermal effect and asymmetric thermal expansion. As a result, the PPy@G‐BC/BOPP actuator, with a bilayer structure and excellent PTE properties, is ideally suited for self‐powered sensing in the form of a light‐driven actuator. As shown in **Figure** **3**a, a test structure that can simultaneously measure the light‐driven and self‐powered sensing properties of the PPy@G‐BC/BOPP actuator was constructed. In order to evaluate the light‐driven properties, a portion (3 cm) of the PPy@G‐BC/BOPP actuator was fixed to a U‐shaped glass frame. While the remaining actuation portion (1.5 cm) was allowed to bend freely under NIR light irradiation. Furthermore, to test the self‐powered sensing properties, one copper electrode was adhered 0.5 cm away from the actuation portion, while the other copper electrode was adhered uppermost for collecting the output thermoelectric signal. Specific experimental details and test structure are given in the Experimental Section and Figure S19 (Supporting Information). The infrared thermal images of the PPy@G‐BC/BOPP actuator under different light power densities are shown in Figure 3b. From these images, it can be seen that the bending angle and the surface temperature of the actuation portion of the PPy@G‐BC/BOPP actuator gradually increase with the enhancement of the light power density. This substantial light‐driven bending is mainly attributed to a combination of two mechanisms. The first mechanism is the thermal‐dehydration effect. Benefiting from its excellent hydrophilicity, the PPy@G‐BC layer can store water molecules in the internal multilayer network structure. When the PPy@G‐BC layer effectively converts light energy into heat energy, the water molecules stored inside will evaporate due to the temperature increase. As a result, the PPy@G‐BC layer will shrink after the water molecules desorb. The second mechanism is the asymmetric thermal expansion effect. Since the PPy@G‐BC/BOPP actuator has a compact bilayer structure, the BOPP layer with a larger CTE will exhibit greater volume expansion than the PPy@G‐BC layer when heated. The volume change mismatch between these two layers will result in internal stresses inside the actuator. The CTE difference is mainly attributed to their different material compositions and structures. The stable multilayer network structure formed by hydrogen bonding and Π‐Π stacking restricts the thermal expansion movement inside the PPy@G‐BC layer upon heating, thus exhibiting a small CTE. In contrast, the polypropylene molecules inside the BOPP layer move more freely during thermal expansion due to the loose structure and weak interaction forces, resulting in a larger CTE. Consequently, a mismatch volume change is caused between the bilayer structure, leading to a substantial bending of the PPy@G‐BC/BOPP actuator toward the BOPP layer.

In order to investigate the correlation between the light‐driven actuation and PTE properties of the PPy@G‐BC/BOPP actuator, Δ*T*, *V*_oc_, and curvature of the PPy@G‐BC/BOPP actuator were simultaneously recorded under different light power densities (Figure S20, Supporting Information). As can be seen, the trends of Δ*T*, *V*_oc_, and curvature of the PPy@G‐BC/BOPP actuator under different light power intensities maintain good consistency. The maximum Δ*T*, maximum *V*_oc,_ and maximum curvature of the PPy@G‐BC/BOPP actuator under different light power densities are exhibited in Figure 3c (data extracted from Figure S20, Supporting Information). It can be seen that the maximum *V*_oc_ and maximum curvature of the PPy@G‐BC/BOPP actuator increased synchronously with the increase in Δ*T*. At a light power density of 300 mW cm^−2^, the PPy@G‐BC/BOPP actuator achieves a maximum curvature of 1.18 cm^−1^. Additionally, the maximum *V*_oc_ and maximum Δ*T* values are 1.127 mV and 27.4 K, respectively. The fitted Sebeck coefficient of the PPy@G‐BC/BOPP actuator is 40.9 µV K^−1^, which is similar to the result of the previous thermoelectric and PTE properties tests (Figure 2d). The above results indicate that the light‐driven deformation hardly interferes with the PTE effect. In other words, the actuation function and the self‐powered sensing function of the PPy@G‐BC/BOPP actuator can be simultaneously effective and unaffected by each other. In addition, a good linear relationship between *V*_oc_ and curvature of the PPy@G‐BC/BOPP actuator (with a slope of 1.08 mV cm^−1^) is demonstrated in Figure S21 (Supporting Information). Based on the excellent linearity, real‐time knowledge of the temperature and deformation state of the PPy@G‐BC/BOPP actuator can be achieved by monitoring the self‐powered output voltage.

Besides the thermoelectric output performance, the photo‐thermal deformation capability is also an important indicator for a light‐driven actuator. Therefore, two flexible photo‐thermal actuation applications have been developed using PPy@G‐BC/BOPP actuators. First, a “panda” robot was created using a PPy@G‐BC/BOPP actuator as a mouth, as shown in Figure 3e. Without light, the PPy@G‐BC/BOPP actuator remained flat, which caused the “Panda” robot to show an indifferent state. As soon as exposed to NIR light, the PPy@G‐BC/BOPP actuator bent drastically due to its excellent photo‐thermal conversion ability, making the “Panda” robot show a happy state. Further, an “athlete” robot was created with PPy@G‐BC/BOPP actuators, which acted as its arms and torso, respectively (Figure 3f). In the absence of light, the “athlete” robot remained flat. Upon activation of the NIR light, the “athlete” robot swiftly executes a push‐up motion, thanks to the rapid photo‐thermal conversion capability of the PPy@G‐BC/BOPP actuators. More importantly, the deformation repeatability of the PPy@G‐BC/BOPP actuator affects its accuracy and reliability for self‐powered sensing applications. Hence, 300 cycles of NIR irradiation (light power density of 200 mW cm^−2^) were performed on the PPy@G‐BC/BOPP actuator. The cyclic test results are displayed in Figure S22 (Supporting Information). After 300 irradiations, there was no significant degradation in the actuation performance of the PPy@G‐BC/BOPP actuator. The maximum curvature at the 300th cycle only decreased by 4.24% compared to the first irradiation. The above results proved that the PPy@G‐BC/BOPP actuator has a long light‐driven service life and stable performance. In the future, PPy@G‐BC/BOPP actuators with sensitive and stable self‐powered sensing and remote actuation capabilities are expected to advance the development of next‐generation multi‐functional actuators.

### 2.4 Self‐Powered Bionic Hand for Intelligent Gesture Recognition

**Figure 4:**
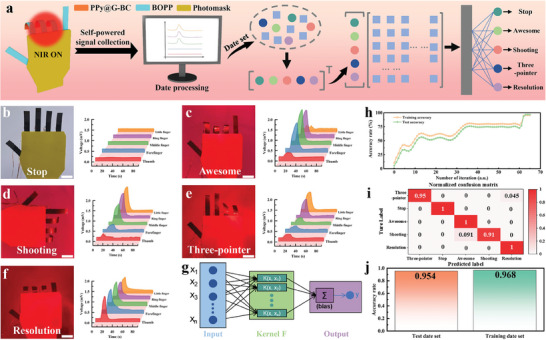
The self‐powered bionic hand for intelligent gesture recognition. a) Schematic flow diagram of gesture recognition. Optical photos and output voltage of the bionic hand with different gestures: b) initial state, c) “Awesome” gesture, d) “Shooting” gesture, e) “Three‐pointer” gesture, f) “Resolution” gesture. Scale bar: 2 cm. g) The typical structure of the machine learning algorithm model: SVM. h) Variation chart of loss and accuracy with the training cycle. i) Confusion matrix of gesture recognition results. j) The accuracy rate of the algorithm model to recognize the gesture data set.

Gesture is a commonly used communication method in daily life that can convey information and express emotions. Inspired by the human five‐fingered hand, a self‐powered bionic hand was designed based on the aforementioned excellent PTE properties of the PPy@G‐BC/BOPP actuator. Five PPy@G‐BC/BOPP actuators were used as bionic fingers by attaching to a palm‐shaped photomask, and the specific dimension of the bionic hand is shown in Figure S23 (Supporting Information). However, the thermoelectric voltage generated by these actuators under different forms of irradiation tends to be complex and variable, which undoubtedly reduces the accuracy of remote gesture recognition. To overcome this problem, we attempted to find a more accurate way to recognize the gestures of the bionic hand. Support vector machine (SVM) algorithm, a classical algorithm in machine learning, is known for its ability to achieve high recognition accuracy with a little amount of training samples. As a result, it is widely used in data analysis and pattern recognition. Therefore, we decided to combine the self‐powered bionic hand with this machine‐learning algorithm to construct an intelligent gesture recognition system. A schematic flow diagram of intelligent gesture recognition is shown in **Figure** **4**a, which incorporates thermoelectric signal collection, data processing, algorithm model training, and gesture recognition results. First, the bionic hand was configured to perform gestures indicating different meanings by using various forms of irradiation while collecting thermoelectric signals generated by these processes. As shown in Figure 4b, when the NIR light was not switched on, the five bionic fingers stayed flat because there was no temperature difference on the surfaces, while the output voltage signals also remained almost unchanged. At this point, the bionic hand exhibited a “Stop” gesture. When the irradiation of NIR light stimulated the bionic forefinger, middle finger, and ring finger, these three bionic fingers were bent at the same time, forming the “Awesome” gesture (Figure 4c). Meanwhile, the self‐powered output voltage signals generated by these three bionic fingers based on the PTE effect followed the same trend as the gesture. In addition, to accomplish other complex gestures such as “Shooting” (left panel of Figure 4d), NIR light was used to irradiate the bionic middle finger, ring finger, and little thumb. And the corresponding electrical signals are shown in the right panel of Figure 4d. Finally, two gestures were designed: the “three‐pointer” gesture with the ring finger and pinky bent (Figure 4e), and the “resolution” gesture with a clenched fist (Figure 4f). The bionic hand also spontaneously generates thermoelectric voltages corresponding to these gestures.

For these five gestures, 50 cycles of thermoelectric signal data were collected, thus creating a database. Then, it was carefully ensured that each sample contained sufficient information. And the collected thermoelectric signals were used as sample features, including the maximum, variance, minimum, deviation, peak, and standard deviation of the total data. As some interference signals occur during the collection of thermoelectric signals, an SVM model was constructed to further process the data, as shown in Figure 4g. Briefly, the principle of this model is to normalize and classify the data, cross‐validate the sample data features after extracting them through the kernel function, and finally pass them to the output layer corresponding to different gestures. As illustrated in Figure 4h, this algorithmic model requires only 65 iterations to obtain a high classification accuracy, which proves its feasibility and accuracy. As can be seen from the confusion matrix for different gestures in Figure 4i, the SVM algorithm has a high recognition rate for the thermoelectric signals of the bionic hand. Specifically, the prediction accuracies of the test set data and the training set data can respectively reach 95.4% and 96.8% (Figure 4j). To summarize, the combination of the self‐powered bionic hand based on PPy@G‐BC/BOPP actuators and the SVM algorithmic model can accurately achieve intelligent gesture recognition, which has a wide range of application prospects in the fields of human‐computer interaction and intelligent recognition.

### 2.5 Humidity‐Driven of PPy@G‐BC/BOPP Actuator

**Figure 5:**
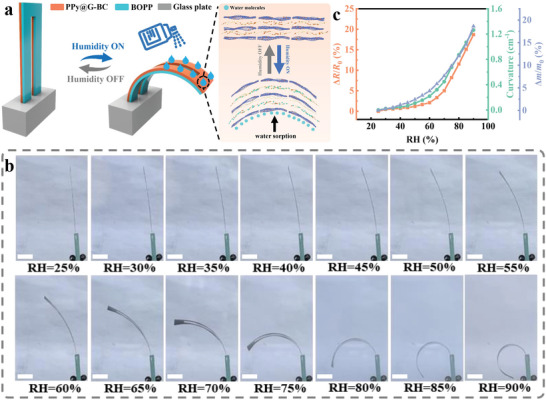
Humidity‐driven actuation and humidity‐sensitive properties of PPy@G‐BC/BOPP actuator. a) Schematic diagram of the humidity actuation. b) Optical photos of the humidity‐driven actuation of the PPy@G‐BC/BOPP actuator. Scale bar: 1 cm. c) Relative resistance change rate, curvature, and mass change of the PPy@G‐BC/BOPP actuator as a function of RH.

As a hydrophilic biomass substrate, BC is highly sensitive to humidity, thanks to the numerous oxygen‐containing groups in its nanofiber network. Therefore, it is reasonable to assume that a PPy@G‐BC/BOPP actuator based on BC nanofibers can be an excellent humidity‐driven actuator. As shown in **Figure** **5**a, a U‐shaped PPy@G‐BC/BOPP actuator with a length of 5 cm was prepared. Specific experimental details and device dimensions are shown in the Experimental Section and Figure S24 (Supporting Information). To investigate the humidity‐driven capability of the PPy@G‐BC/BOPP actuator, it was placed in a humidity‐controlled chamber with a relative humidity (RH) of 25% and humidified. As can be seen in the optical photos of Figure 5b, the actuator exhibited substantial bending actuation during humidification, with RH ranging from 25% to 90%. When the RH was 90%, the maximum bending curvature of the PPy@G‐BC/BOPP actuator was up to 1.26 cm^−1^, demonstrating its excellent humidity‐driven capability. The mechanism of humidity‐driven actuation of the PPy@G‐BC/BOPP actuator can be explained by the asymmetric humidity expansion effect between the PPy@G‐BC layer and the BOPP layer (Figure 5a). First, as can be seen from the hydrophilicity test in Figure S25a–c (Supporting Information), the PPy@G‐BC film exhibits a small water contact angle (WCA) of ≈37.9°, which is attributed to the large number of hydrophilic functional groups (e.g., ‐OH and ‐COOH) inside. Not only that, the multilayer compact structure inside the PPy@G‐BC film also contributes to the absorption and storage of water molecules. Although the concave‐convex surface structure formed by the polymerization of PPy nanoparticles resulted in a small deterioration in the hydrophilicity of the PPy@G‐BC film compared to the G‐BC film (WCA of 33.7°), the proper hydrophilicity can prevent the actuator from excessive humidity damage. In contrast, the large WCA of 71.6° for BOPP film proves it to be a highly hydrophobic polymer material, which is inert to external humidity changes. Hence, there is a huge asymmetry in the water absorption capacity between the PPy@G‐BC layer and the BOPP layer. The above analysis can be proved by the results of the water absorption tests in Figure S25d (Supporting Information). The mass change of the PPy@G‐BC film is over 20% when the RH increases from 25% to 90%, while that of the BOPP film is only 4%. Therefore, when the environmental humidity increases, the physical morphology of the BOPP layer remains almost unchanged, while the PPy@G‐BC layer absorbs water and expands. As a result, the PPy@G‐BC layer and the BOPP layer exhibit asymmetric humidity volume expansion in a humid environment. The expansion difference generates internal stresses that cause the actuator to bend toward the BOPP layer with less volume change.

Further, the relative resistance change rate, curvature, and mass change of the PPy@G‐BC/BOPP actuator under different humidity conditions were comprehensively analyzed to investigate the humidity sensitivity of the PPy@G‐BC/BOPP actuator. The corresponding curves of relative resistance change rate, curvature, and mass change are recorded in Figure 5c. In an environment with an initial RH of 25%, the PPy@G‐BC/BOPP actuator stayed flat while its resistance remained almost constant. As the curvature of the PPy@G‐BC/BOPP actuator increased to 1.26 cm^−1^, its maximum relative resistance change rate reached 18.7% at the RH of 90%. It is because when the PPy@G‐BC layer absorbs water and expands, the electron transfer efficiency inside will be hindered by the water molecules with higher resistivity, which in turn increases the overall resistance. The trends of curvature, relative resistance change rate, and mass change of the PPy@G‐BC/BOPP actuator exhibit good consistency with the change of RH. Therefore, the water content change of the PPy@G‐BC/BOPP actuator can be monitored by analyzing the relative resistance change rate.

### 2.6 Self‐Powered Humidity Sensing Function of PPy@G‐BC/BOPP Actuator

**Figure 6:**
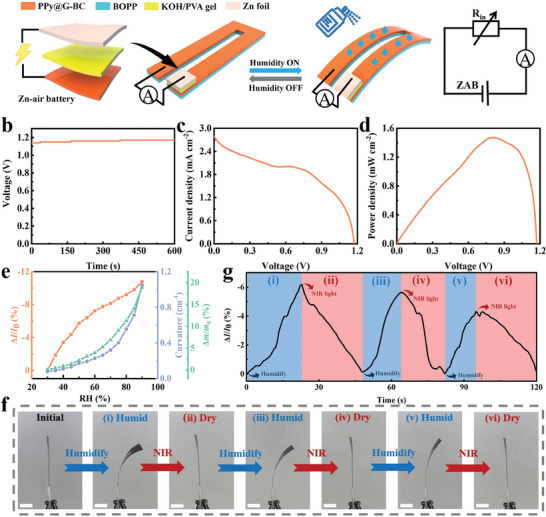
Humidity actuation and self‐powered humidity sensing of PPy@G‐BC/BOPP actuator. a) Schematic diagram of the structure of the PPy@G‐BC/BOPP actuator in situ integrated with zinc‐air battery. b) The open‐circuit voltage generated by the zinc‐air battery based on PPy@G‐BC film. c) The current density of the zinc‐air battery based on PPy@G‐BC film. d) The power density of the zinc‐air battery based on PPy@G‐BC film. e) The relative current change rate, curvature, and mass change of the integrated PPy@G‐BC/BOPP actuator. f) Optical photos of the deformation of the PPy@G‐BC/BOPP actuator under light/humidity dual stimulation. Scale bar: 1 cm. g) Performance of PPy@G‐BC/BOPP actuator under light/humidity dual stimulation.

Although the water content of the PPy@G‐BC/BOPP actuator can be monitored by analyzing the relative resistance change rate, the complex external power supply and connecting wires would inevitably complicate the sensing system. With the development of flexible electronic devices, flexible zinc‐air batteries have become a prospective renewable energy solution due to their high energy density, low cost, and environmental friendliness.^[^ ^23^, ^48^ ^]^ Inspired by this, we attempted to integrate a flexible zinc‐air battery in situ on the PPy@G‐BC layer as an energy module of the PPy@G‐BC/BOPP actuator, as shown in **Figure** **6**a. Thanks to the excellent conductivity of the PPy@G‐BC layer, the in‐situ integration process of the zinc‐air battery does not require any wires, showing a high degree of integration and flexibility. Specific experimental details and device structures are described in the Experimental Section and Figure S26 (Supporting Information). The flexible zinc‐air battery is a battery that generates electrical energy through redox reactions. It has a semi‐open structure composed primarily of a zinc anode, an air cathode (PPy@G‐BC layer), and an alkaline solid electrolyte (KOH/PVA gel electrolyte). The specific electrochemical reactions during the discharge process are detailed in Note S2 (Supporting Information). Next, a 6 M KOH/PVA gel was used as a solid‐state electrolyte to test the performance of PPy@G‐BC film in a solid‐state zinc‐air battery, as shown in Figure 6b–d. The results indicate that the solid‐state zinc‐air battery also possesses stable output performance, where the maximum open‐circuit voltage, maximum current density, and maximum power density can respectively reach 1.17 V, 2.79 mA cm^−2^, and 1.47 mW cm^−2^.

As can be seen in Figure S27 (Supporting Information), the humidity‐driven PPy@G‐BC/BOPP actuator integrated with a zinc‐air battery can still perform substantial humidity‐driven deformation. The maximum relative current change rate and the maximum curvature of the PPy@G‐BC/BOPP actuator can reach 10.76% and 1.05 cm^−1^, respectively (Figure 6e). Notably, the water content remains well consistent with the curvature but has a symmetric relationship with the current rate of change. This phenomenon is because as the output voltage of the integrated zinc‐air battery remains constant, the overall resistance of the device increases due to the water absorption in the PPy@G‐BC/BOPP actuator. As a result, the current change rate increased nonlinearly. We further investigated the capability of PPy@G‐BC/BOPP actuators to detect external environmental changes under light/humidity dual stimulation. Optical photos of the experimental process are shown in Figure 6f. The PPy@G‐BC/BOPP actuator was humidified to different bending levels and then dried to a straight state through NIR light while collecting current signals during the process. It can be seen from Figure 6g that the relative current change rate of the PPy@G‐BC/BOPP actuator varies synchronously with the deformation generated by humidification and irradiation. In addition, the PPy@G‐BC/BOPP actuator was also tested with cyclic light/humidity dual stimulation, as shown in Figure S28 (Supporting Information). As can be seen, the PPy@G‐BC/BOPP actuator exhibited stable light/humidity‐driven deformation performance and self‐powered sensing performance in the cyclic test, which indicates its combination of flexibility and durability. The above results indicate that the PPy@G‐BC/BOPP actuator integrated with a zinc‐air battery has excellent self‐powered humidity sensing capabilities. The novel self‐powered device with a multi‐mode sensing function will provide new ideas for the development of the environmental monitoring field.

### 2.7 Intelligent Gripper Integrated with Multi‐Mode and Self‐Powered Sensing Function

**Figure 7:**
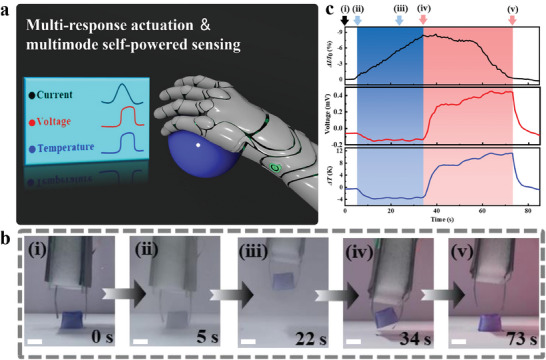
The intelligent gripper with self‐powered multi‐mode sensing function. a) Schematic diagram of the intelligent gripper gripping an object while realizing self‐powered multi‐mode sensing. b) A series of optical photos of the intelligent gripper during the operating process. Scale bar: 1 cm. c) Relative current change rate, output voltage, and temperature difference of the PPy@G‐BC/BOPP actuators in the gripper during the operating process (corresponding to Figure 7(b)).

Under different environmental stimuli, the light/humidity‐driven actuators based on PPy@G‐BC film have the capability of spontaneously outputting electrical signals during the actuation deformation process. Based on these advantages, a PPy@G‐BC/BOPP actuator with a self‐powered temperature sensing function and a PPy@G‐BC/BOPP actuator with a self‐powered humidity sensing function were highly integrated into a single intelligent gripper (**Figure** **7**a). The structure of the intelligent gripper is schematically shown in Figure S29 (Supporting Information). The strip‐shaped PPy@G‐BC/BOPP actuator and the U‐shaped PPy@G‐BC/BOPP actuator were placed face‐to‐face. The specific preparation method of the intelligent gripper is detailed in the Experimental Section. The optical photos of the movement process of the intelligent gripper are shown in Figure 7b. As shown in Figure 7c, when there was no external stimulus, the intelligent gripper did not exhibit any deformation or electrical signal changes (Figure 7b(i)). After applying the humidity stimulus, the actuators on both sides of the intelligent gripper started to bend toward the object (Figure 7b(ii)) and grasped the object to perform lifting and lowering operations (Figure 7b(iii),(iv)). Notably, the weight of the grasped object is approximately twice the weight of the actuation parts of these two actuators. During the process, the current generated by the PPy@G‐BC/BOPP actuator decreased rapidly (blue part of the upper panel in Figure 7c). Notably, the voltage generated by the PPy@G‐BC/BOPP actuator also decreased slightly (blue region of the middle panel in Figure 7c). It is because the PPy@G‐BC/BOPP actuator sensed a small temperature drop caused by the humidity stimulus. When the humidity stimulus stopped and the NIR light stimulus was simultaneously turned on, the actuation portions of the intelligent gripper gradually heated up by the irradiation. Finally, the intelligent gripper slowly reverted to its initial deformation state, and the object was released (Figure 7b(v)). During the release process, the current generated by the PPy@G‐BC/BOPP actuator increased slowly due to the water loss of the actuator under irradiation (red region of the upper panel in Figure 7c). The voltage generated by the PPy@G‐BC/BOPP actuator significantly increased due to the heating of the actuator (red region of the middle panel in Figure 7c). From the above results, it can be found that there is a good correspondence between the working state of the intelligent gripper, the electrical signals, and the temperature difference change (the bottom panel in Figure 7c). It indicates that the intelligent gripper can remotely realize self‐powered and multi‐mode (temperature/humidity) sensing while grasping the object. In summary, the ingenious strategy of integrating multi‐responsive actuation, self‐powered, and multi‐mode sensing allows the full utilization of multiple environmental stimuli as well as the complementary synergistic monitoring of multiple physical properties.

## 3 Conclusion

In summary, the free‐standing PPy@G‐BC film with a multilayer network structure was easily fabricated by a low‐cost method combining vacuum filtration and in‐situ polymerization. The strong physical and chemical cross‐linking between graphene, BC, and PPy endows the PPy@G‐BC film with excellent tensile strength (93.53 MPa) and Young's modulus (5.55 GPa), which is an important basis for the actuation deformation. Moreover, the PPy@G‐BC film possesses good PTE properties as a photo‐thermal layer (Seebeck coefficient of 42.8 µV K^−1^) and good hydrophilicity as a humidity‐sensitive layer (WAC of 37.9°). Based on these excellent comprehensive properties of the PPy@G‐BC film, two types of actuators with self‐powered and multi‐mode sensing functions were developed. One is the light‐driven PPy@G‐BC/BOPP actuator that deforms based on the asymmetric thermal expansion effect. The PPy@G‐BC/BOPP actuator can spontaneously generate thermoelectric voltage, thus reflecting its own deformation state and temperature changes in real‐time. A self‐powered bionic hand was designed based on this light‐driven actuator, which can perform various complex gestures. With the assistance of SVM algorithms, various gestures made by this bionic hand can be accurately recognized with an accuracy of 96.8%. The other is the humidity‐driven PPy@G‐BC/BOPP actuator that deforms based on the asymmetric humidity expansion effect. The PPy@G‐BC/BOPP actuator realizes self‐powered monitoring of its own deformation state and humidity by in situ integrating a flexible zinc‐air battery. The actuation deformation and self‐powered sensing functions of the two aforementioned actuators are in good consistency without interfering with each other. Finally, with the complementary synergy of these two actuators, the intelligent gripper can not only move objects under the dual stimulation of light and humidity but also realize the multi‐mode (temperature/humidity) sensing function. The ingenious strategy of simultaneously realizing multi‐responsive actuation and self‐powered sensing in a single device will advance the fields of flexible electronics, soft robots, human‐machine interaction, and environmental monitoring.

## Conflict of Interest

The authors declare no conflict of interest.

## Supporting information

Supporting Information

## Data Availability

The data that support the findings of this study are available from the corresponding author upon reasonable request.
