# Supplementary material for: Multi‐Functional Actuators Made with Biomass‐Based Graphene‐Polymer Films for Intelligent Gesture Recognition and Multi‐Mode Self‐Powered Sensing
Source: Adv Sci (Weinh). 2024 Mar 26;11(22):2309846. doi: 10.1002/advs.202309846 (PMC11165533; doi:10.1002/advs.202309846)
Supplement: Supplementary file 1 — Supporting Information [file ADVS-11-2309846-s001.pdf]

ADVANCED SCIENCE
Open Access

Supporting Information

for *Adv. Sci.*, DOI 10.1002/advs.202309846

Multi-Functional Actuators Made with Biomass-Based Graphene-Polymer Films for Intelligent Gesture Recognition and Multi-Mode Self-Powered Sensing

Mingcen Weng, Jiahao Zhou, Peidi Zhou, Ruzhi Shang, Minghua You, Guozhen Shen* and Huamin Chen**

Supporting Information

Multi-functional Actuators Made with Biomass-based Graphene-polymer Films for Intelligent Gesture Recognition and Multi-mode Self-powered Sensing

Mingcen Weng, Jiahao Zhou, Peidi Zhou, Ruzhi Shang, Minghua You, Guozhen Shen*, Huamin Chen**

Experimental Section

1 Material:

Bacterial cellulose nanofibers (average diameter of 75 nm, average length of 20 μm) were purchased from Guilin Qihong Technology Co., Ltd. Aqueous graphene paste was purchased from Nacate New Material Technology Co., Ltd. Both Pyrrole and iron chloride hexahydrate were provided by Shanghai Macklin Biochemical Co., Ltd. The BOPP film (coated with acrylic ester) was a commercial product with a thickness of 38 μm. Potassium hydroxide is purchased from Sinopharm Chemical Reagent Co., Ltd. Polyvinyl alcohol powder was purchased from Shanghai Aladdin Biochemical Technology Co., Ltd. High purity zinc foil purchased from Weng Hou Metal Material Trading Company in Shushan District, Hefei City. These products can be used upon receipt and do not require further purification.

2 Preparation of G-BC film

The G-BC films were prepared by a facile vacuum filtration method. A high weight ratio of graphene will lead to the deterioration of mechanical properties and hydrophilicity of the composite films. While a low weight ratio of graphene will affect the electrical properties, photo-thermal conversion performance, and thermoelectric properties of the composite films. Therefore, 1:1 was chosen as the weight ratio of graphene to BC. First, aqueous graphene pastes and BC nanofibers were added to deionized water and then magnetically stirred for 15 min to obtain the G-BC solution. Next, the G-BC solution was sonicated for 15 min to obtain a uniformly dispersed G-BC suspension. Finally, the G-BC film was easily obtained by filtering the G-BC suspension through a filter membrane and drying it at 40 °C for 2 h.

3 Preparation of PPy@G-BC/BOPP actuator

PPy@G-BC films were prepared by a simple in situ polymerization method. First, pyrrole was

added to ethanol solution to configure 0.2 mol/L pyrrole solution, and iron chloride hexahydrate was added to ethanol solution to configure 0.2 mol/L $FeCl_3$ solution. Then, the above pyrrole solution and iron chloride hexahydrate solution were added successively to the petri dish in which the G-BC composite was placed. Then, the petri dish was placed in a refrigerator at 5 ℃ for 12 h to obtain the PPy@G-BC film with in-situ polymerized polypyrrole nanoparticles. Then, after careful rinsing with alcohol and deionized water in turn, the polymerized PPy@G-BC film was dried at 40 °C for 2 h. The thickness of the PPy@G-BC film was 26 μm. Finally, the BOPP film was tightly adhered to the PPy@G-BC film through acrylic ester to obtain a PPy@G-BC/BOPP actuator with a bilayer structure. The thickness of the PPy@G-BC/BOPP actuator was about 64 μm.

4 TE property test

First, a PPy@G-BC film with dimensions of 3 cm × 1 cm (length × width) was prepared. Then, two copper foils were embedded into the ends of the PPy@G-BC film through silver glue as electrodes (the position of the copper foils is shown in Figure 2a and Figure S10). Finally, the PPy@G-BC film was placed between the two platforms, with a length of 1 cm in the middle overhang. In the TE test, the cold platform was used as the cold end, and the hot platform was used as the hot end. The left and right ends of the PPy@G-BC film (both 1 cm in length) were fixed to the hot platform and the cold platform with polyimide tape. During the heating process of the hot platform, the temperature difference and the open circuit voltage (V_{oc}) between the two electrodes of the PPy@G-BC film were measured simultaneously. I_{sc} was measured during the same heating process. ΔT is defined as the temperature difference between the two electrodes of the PPy@G-BC film.

5 PTE property test

First, a U-shaped glass frame was prepared, and a BOPP film was attached to the PPy@G-BC

film with dimensions of 3.5 cm × 1 cm (length × width). Then, two copper foils were embedded into the ends of the PPy@G-BC/BOPP actuator as electrodes, and the position of the copper foils is shown in Figure S16. Finally, a copper tape with a length of 2.5 cm was pasted on the PPy@G-BC/BOPP actuator, leaving an irradiated portion with a length of 1 cm. During the irradiation of NIR light with different power densities (50, 100, 150, 200, 250, and 300 mW cm^{-2}), the temperature difference and V_{oc} between the two electrodes of the PPy@G-BC/BOPP actuator were measured simultaneously.

6 Light-driven actuation and self-powered sensing test

First, a U-shaped glass frame was prepared. Then, a BOPP film was attached to a PPy@G-BC film with dimensions of 4.5 cm × 1 cm (length × width). The lengths of the non-deformable part and the free bending part of the PPy@G-BC/BOPP actuator were 3 cm and 1.5 cm, respectively. Then, a copper tape with a length of 2.5 cm was pasted on the PPy@G-BC/BOPP actuator as a photomask, leaving an irradiated portion with a length of 2 cm. Finally, two copper foils were embedded into the ends of the PPy@G-BC/BOPP actuator as electrodes, and the position of the copper foils is shown in Figure 3a and Figure S19. During the irradiation of NIR light with different power densities (50, 100, 150, 200, 250, 300 mW cm^{-2}), the temperature difference and V_{oc} between the two electrodes of the PPy@G-BC/BOPP actuator were measured simultaneously. At the same time, the bending deformation of the PPy@G-BC/BOPP actuator was recorded in real time by a smartphone.

7 Preparation of light-driven bionic hand

First, a bilayer copper foil paper was sandwiched between two palm-shaped black cardboard to form a photomask. Then, copper foils were embedded in five PPy@G-BC/BOPP actuators through silver glue. Finally, five PPy@G-BC/BOPP actuators were attached in position to the palm-shaped photomask to form the bionic five-fingered palm. The exact position and

dimensions of the films and the copper foils embedded in them are shown in Figure S23. The V_{oc} of the PPy@G-BC/BOPP actuator was recorded in real time during the light-driven process.

8 Preparation of KOH/PVA gel electrolyte

First, 30 mL deionized water was preheated to 90 °C. Then, 3 g polyvinyl alcohol (PVA) powder was added to the preheated deionized water in three portions and mixed under strong magnetic stirring until transparent. Next, the ground KOH powder was added to the PVA gel solution in three portions and mixed under strong magnetic stirring until the mixed gel solution was transparent and pale yellow in color. Then, the mixed gel solution was poured into a petri dish and frozen at -40 °C for 12 h followed by thawing at 25 °C for 2 h for three cycles. Finally, the prepared KOH/PVA gel sheet was peeled off the petri dishes to obtain a flexible gel electrolyte.

9 Humidity-driven actuation and humidity-sensitive properties test

First, a PPy@G-BC/BOPP actuator with dimensions of 5 cm × 1 cm (length × width) was prepared under 25% RH. Then, the middle part of the PPy@G-BC/BOPP actuator with a size of 4.5 cm × 0.2 cm (length × width) was cut off, and the resulting U-shaped actuator was shown in Figure 5a and Figure S24. Finally, two copper foils were attached to the two ends of the arms of the U-shaped PPy@G-BC/BOPP actuator as electrodes through silver glue. In a self-made humidity-controlled chamber, the change of the room RH from 25% to 90% was controlled by a dehumidifier and humidifier in cooperation. During the RH change, the room RH and the resistance of the PPy@G-BC/BOPP actuator were recorded simultaneously. At the same time, the bending deformation of the PPy@G-BC/BOPP actuator was recorded in real time by a smartphone.

10 Humidity-driven actuation and self-powered humidity sensing test

First, a PPy@G-BC/BOPP actuator with dimensions of 5 cm × 1 cm (length × width) was cut

into a U-shape. Then, a KOH/PVA gel electrolyte and flexible zinc foil with the same dimensions of 0.5 cm × 0.5 cm were integrated in-situ into the end of a single arm of the U-shaped PPy@G-BC/BOPP actuator as a power supply unit. Then, two copper foils were attached to the zinc foil and the end of the other arm of the actuator through silver glue as electrodes, respectively. Finally, the zinc-air battery was encapsulated through BOPP films to obtain a humidity-driven actuator with self-powered sensing function. The detailed shape and dimensions of this actuator are shown in Figure 6a and Figure S26. During humidity changes, the RH of the room and the output current signal of the PPy@G-BC/BOPP actuator were recorded simultaneously. At the same time, the bending deformation of the PPy@G-BC/BOPP actuator was recorded in real time through a smartphone.

11 Preparation of the intelligent gripper integrated with self-powered sensing function

As shown in Figure 7a and Figure S29, the rectangular PPy@G-BC/BOPP actuator and the U-shaped PPy@G-BC/BOPP actuator were fixed to two U-shaped frames, respectively. The rectangular PPy@G-BC/BOPP actuator was used as a light-driven actuator for self-powered temperature monitoring, and the U-shaped PPy@G-BC/BOPP actuator was used as a humidity-driven actuator for self-powered humidity monitoring. And the copper tape as a photomask was placed 2.5 cm away from the tail of the PPy@G-BC/BOPP actuator. These two PPy@G-BC/BOPP actuators were placed face-to-face in a symmetrical configuration to form an intelligent gripper integrated with self-powered sensing functions. When the NIR light was switched on, only the tail (2 cm) of the PPy@G-BC/BOPP actuators would bend from irradiation. The weight of the object grasped by the intelligent gripper was 35 mg. While, the weights of the actuation parts of the PPy@G-BC/BOPP light-driven actuator and the PPy@G-BC/BOPP humidity-driven actuator were approximately 18 mg and 15 mg, respectively.

12 Characterization and measurements

The morphologies and microstructures of the materials were characterized with a transmission electron microscope (TEM) (JEM-2100) and a scanning electron microscope (SEM) (SU8000, JPN). Their molecular compositions and phase structures were identified with a Fourier transform infrared spectrometer (FTIR) (Thermo Fisher, Nicolet 6700, USA) and an X-ray diffractometer (XRD) (Bruker Corporation, D8 advance, GER). The tensile properties of the samples were obtained using a universal testing machine (Instron, 3343). The temperature of the hot and cold ends of the materials in the TE test was recorded by a digital thermometer (UNI-T, UT325). Thermal conductivity was measured by Hot Disk thermal constant analyzer (TPS2500S). Light power density was measured by an infrared power meter (Linshang, LS122). The surface temperature and heat distribution of the materials were recorded by using an infrared thermometer (Optris, MS Pro) with a temperature resolution of 0.1 °C and an infrared thermal camera (Hikvision, H16), respectively. A benchtop digital multimeter (UNI-T, UT805A+) was used to record electrical signals. The RH was recorded by a digital hygrometer (UNI-T, UT332+). Optical photos and videos were recorded by a smartphone (iPhone 12).

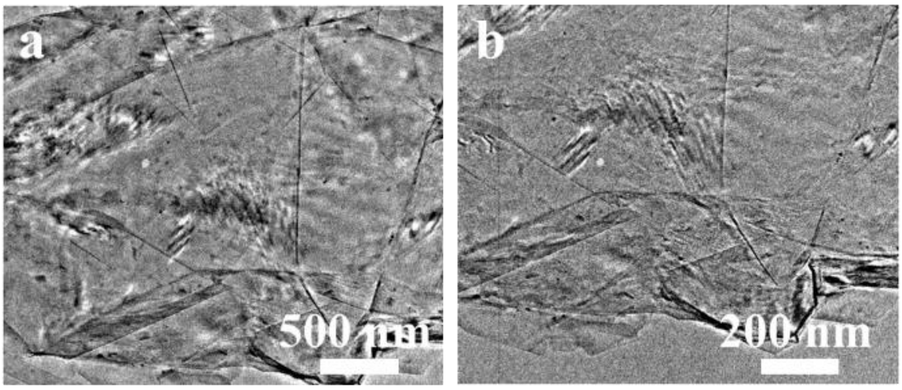

Figure S1. TEM images of the graphene nanosheets at different magnifications.

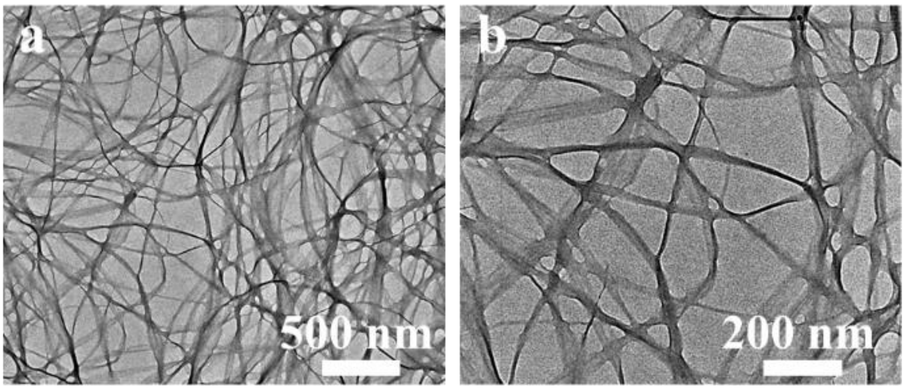

Figure S2. TEM images of the BC nanofibers at different magnifications.

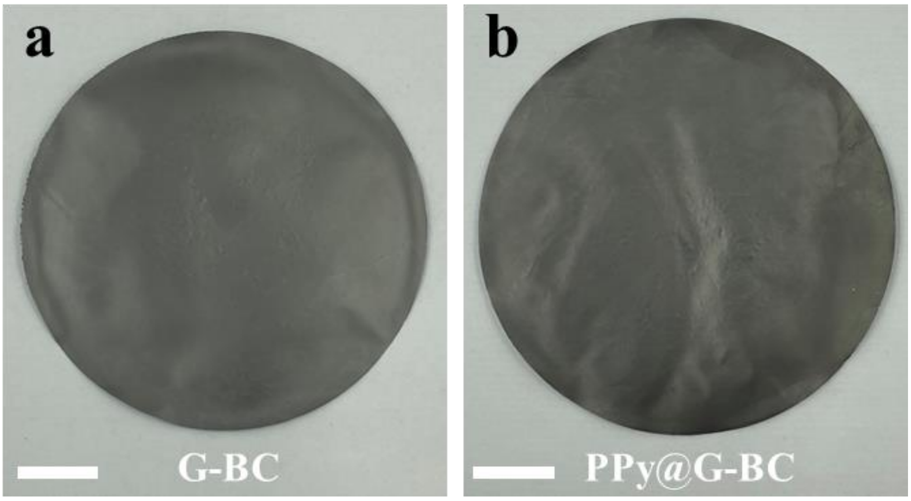

Figure S3. (a) Optical photo of the G-BC film. (b) Optical photo of the PPy@G-BC film. Scale bar: 2 cm.

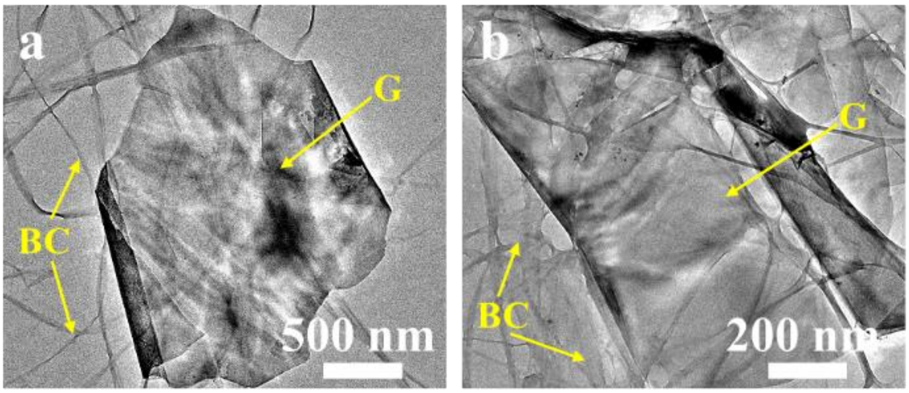

Figure S4. TEM images of the G-BC composite at different magnifications.

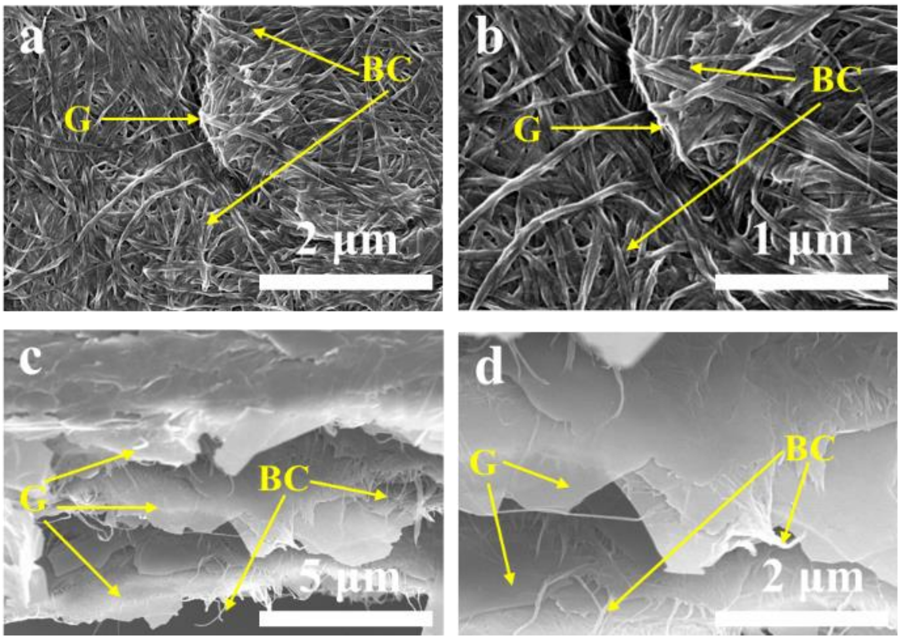

Figure S5. (a)-(b) SEM images of the surface of the G-BC film at different magnifications. (c)-(d) SEM images of the cross section of the G-BC film at different magnifications.

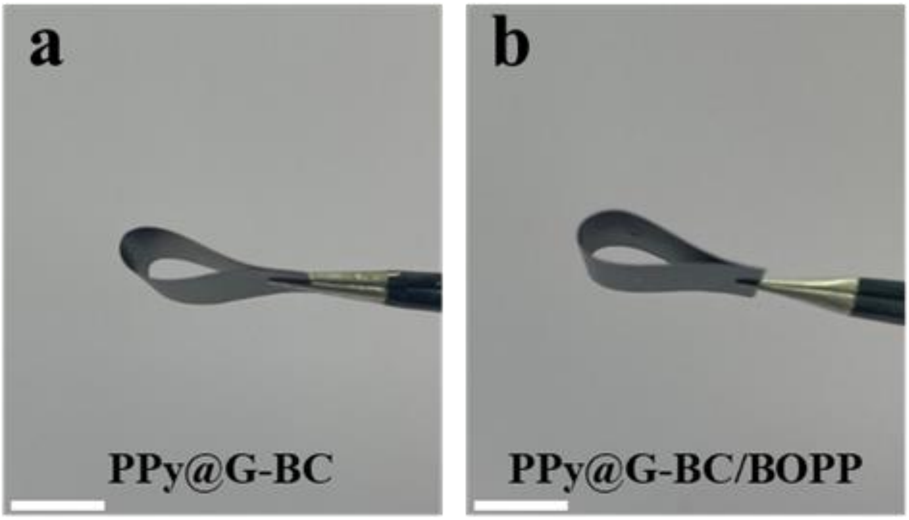

Figure S6. (a) Optical photo of the G-BC film in the bending state. (b) Optical photo of the PPy@G-BC film in the bending state. Scale bar: 1 cm.

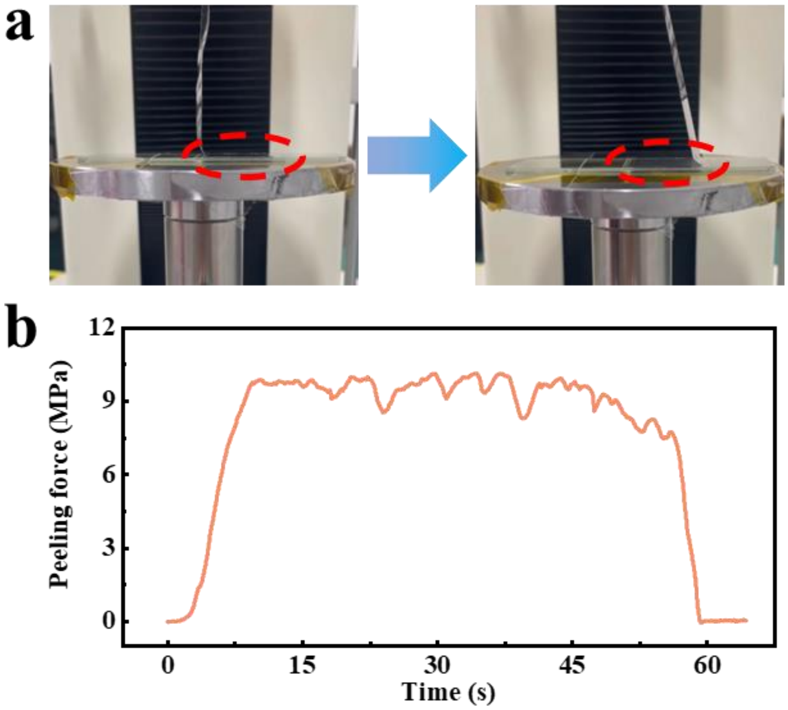

Figure S7. (a) Optical photos of the PPy@G-BC/BOPP actuator peeling experiment. (b) Peeling force of the PPy@G-BC/BOPP actuator.

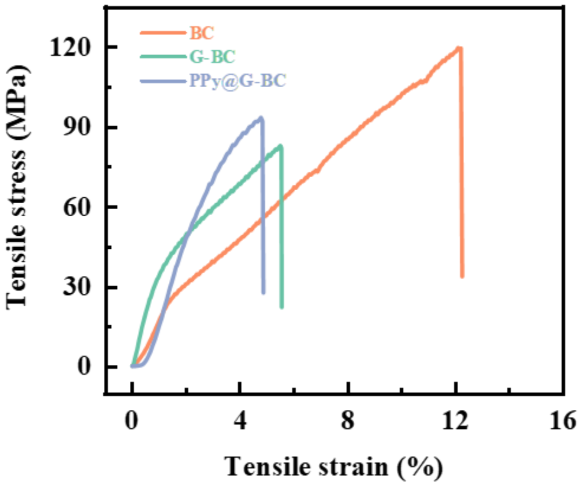

Figure S8. Tensile stress-strain curves of the BC film, the G-BC film, and the PPy@G-BC film.

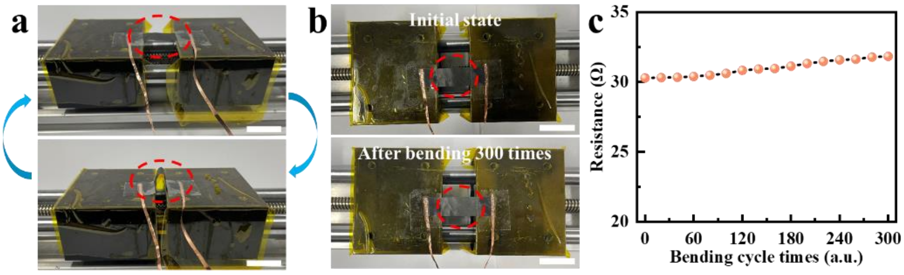

Figure S9. Mechanical stability of PPy@G-BC film. (a) Optical photos of the PPy@G-BC film in bending cycle test. (b) optical photos of the PPy@G-BC film surface between the initial state and after 300 bending cycles. (c) Resistance of PPy@G-BC film in bending cycle test.

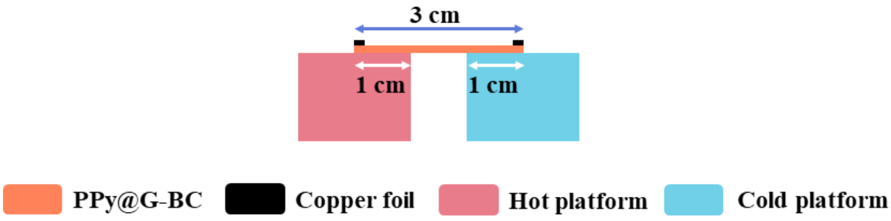

Figure S10. Dimensions of the PPy@G-BC film when heated by a hot platform.

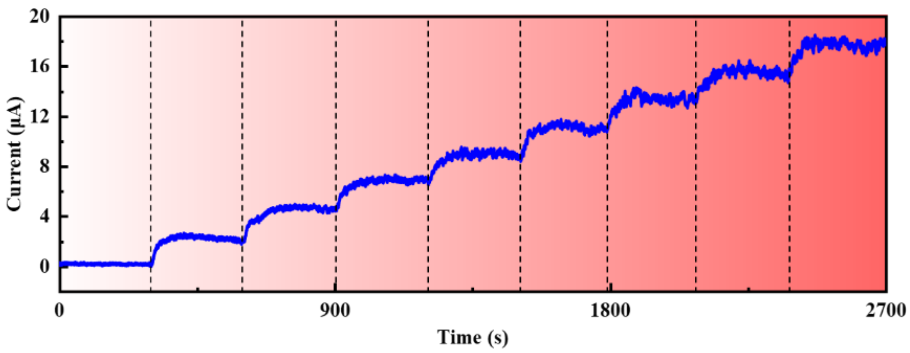

Figure S11. I_{sc} of the PPy@G-BC film when heated by a hot platform.

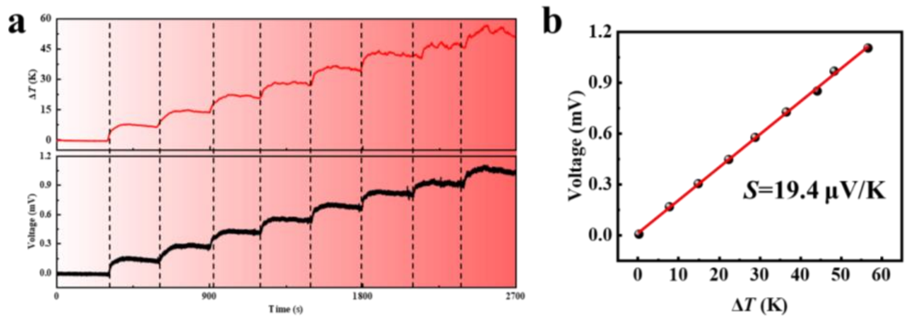

Figure S12. TE property of G-BC film. (a) ΔT and V_{oc} of the G-BC film heated by a hot plate. (b) Output voltage of the G-BC film as a function of ΔT.

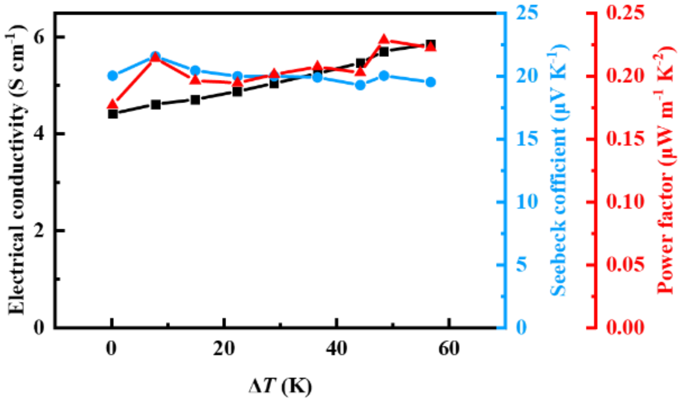

Figure S13. Electrical and thermoelectric properties of the G-BC film under different temperature differences.

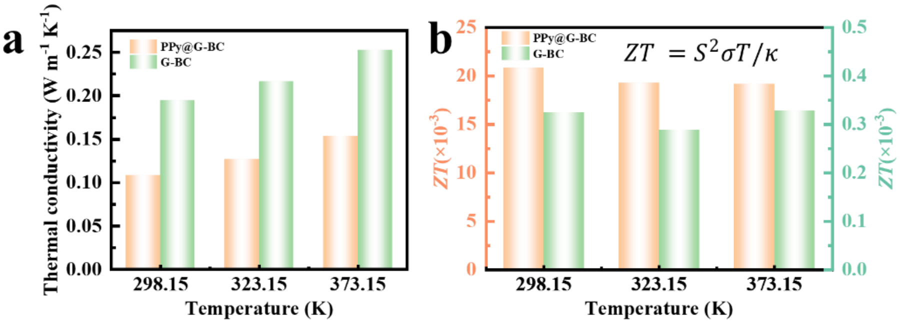

Figure S14. (a) Comparison of thermal conductivity of the PPy@G-BC film and the G-BC film. (b) Comparison of *ZT* values of the PPy@G-BC film and the G-BC film.

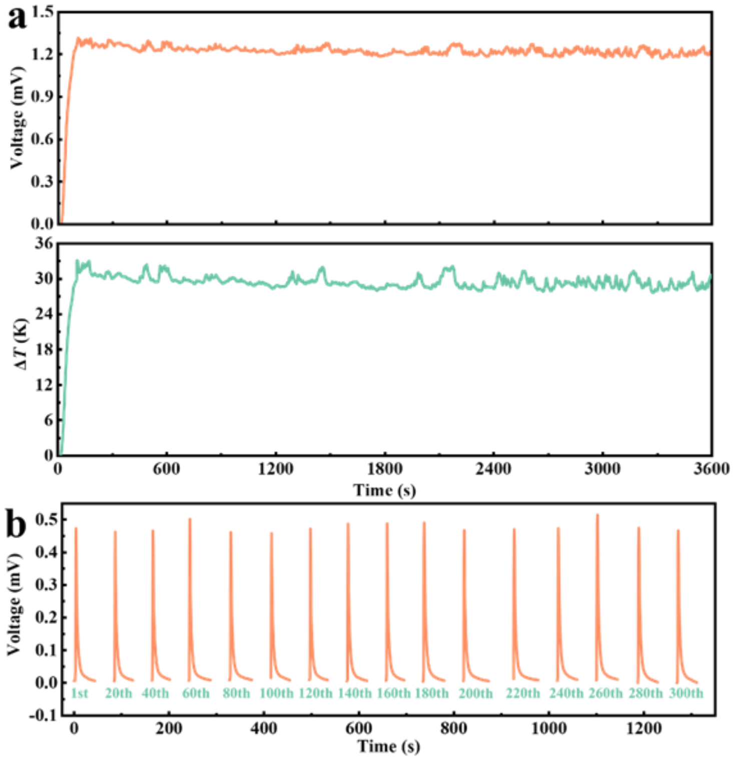

Figure S15. (a) Thermoelectric stability test of PPy@G-BC film (b) Thermoelectric durability test of the PPy@G-BC film.

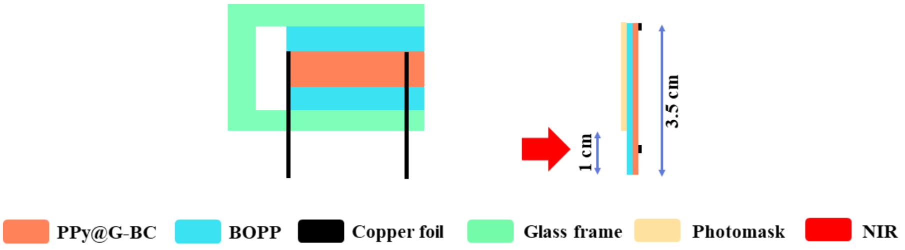

Figure S16. Dimensions of the PPy@G-BC/BOPP actuator when irradiated by NIR light.

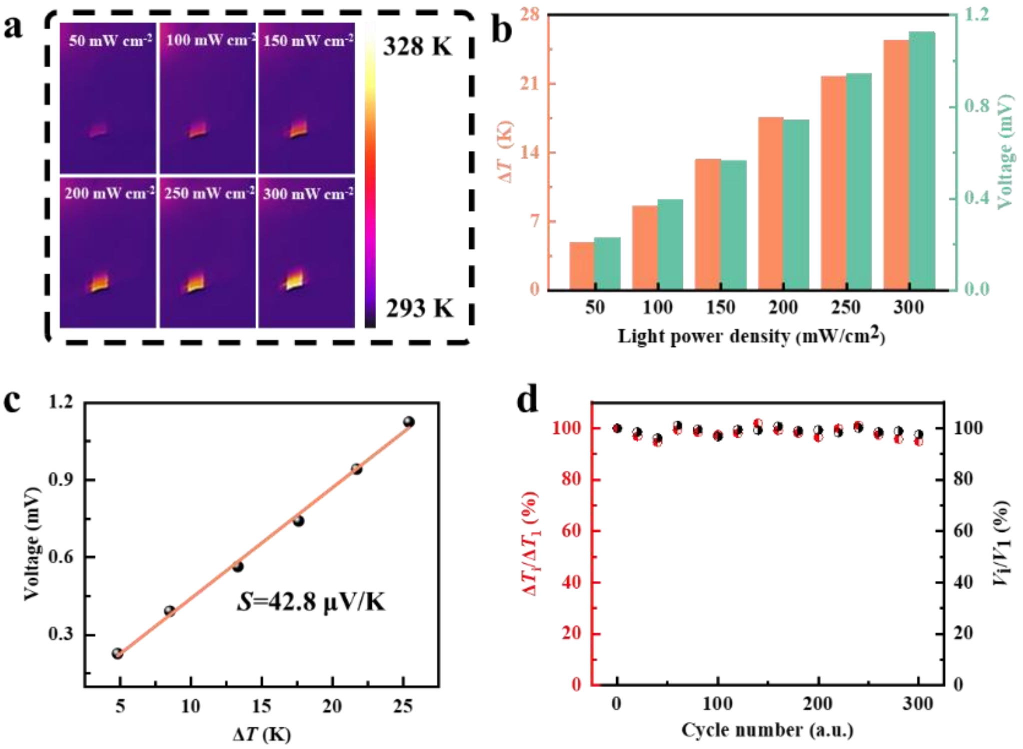

Figure S17. PTE property of PPy@G-BC/BOPP actuator. (a) Infrared thermal images of the PPy@G-BC/BOPP actuator under different light power densities. (b) ΔT and V_{oc} of the PPy@G-BC/BOPP actuator under different light power densities. (c) Output voltage of the PPy@G-BC/BOPP actuator as a function of ΔT when irradiated by NIR light. (d)Changes in temperature and output voltage of PPy@G-BC/BOPP actuator over 300 cycles (light power density of 200 mW cm^{-2}).

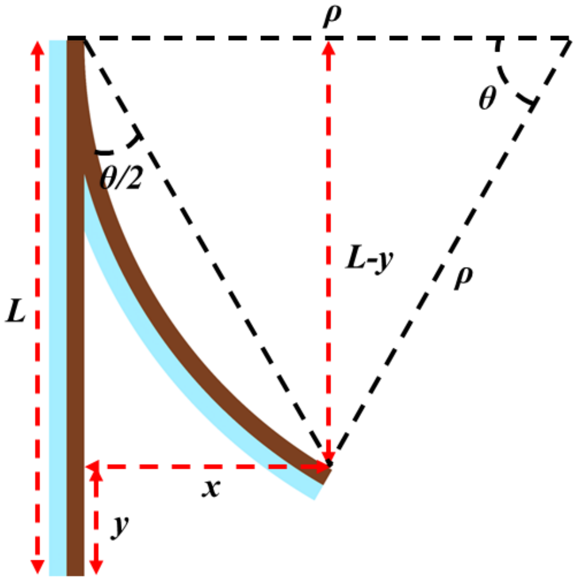

Figure S18. Actuation part of actuator with correlative parameters for calculating the bending curvature.

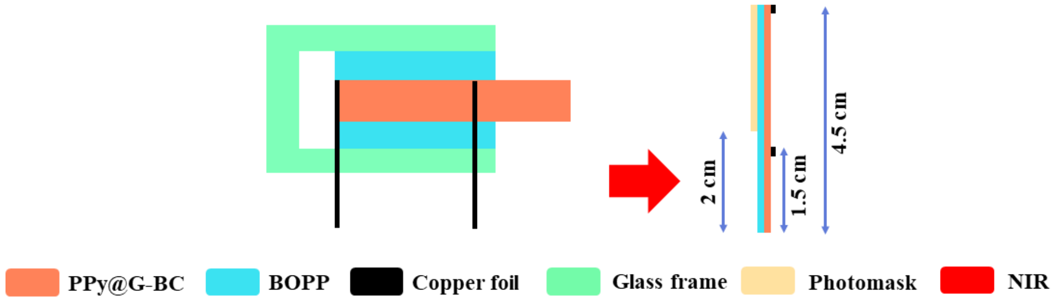

Figure S19. Dimensions of the PPy@G-BC/BOPP actuator when irradiated by NIR light.

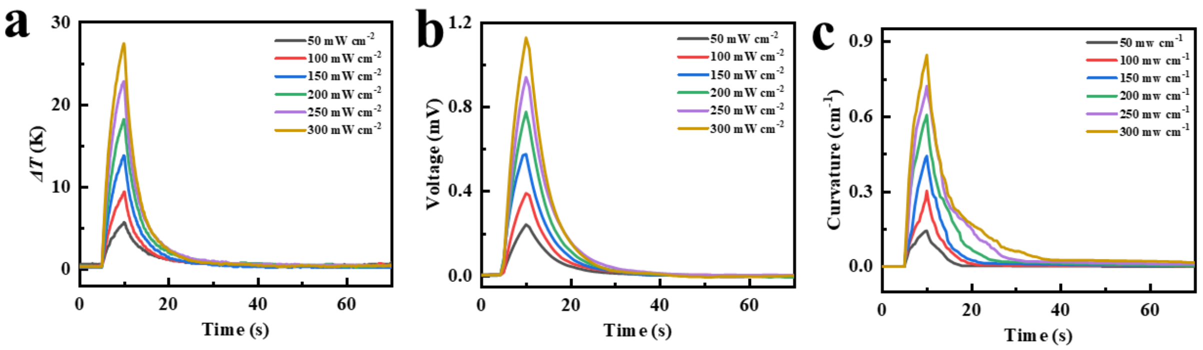

Figure S20. (a) Temperature difference variation of the PPy@G-BC/BOPP lihgt-driven actuator under different light power densities. (b) Output voltage variation of the PPy@G-BC/BOPP actuator under different light power densities. (c) Curvature variation of the PPy@G-BC/BOPP actuator under different light power densities.

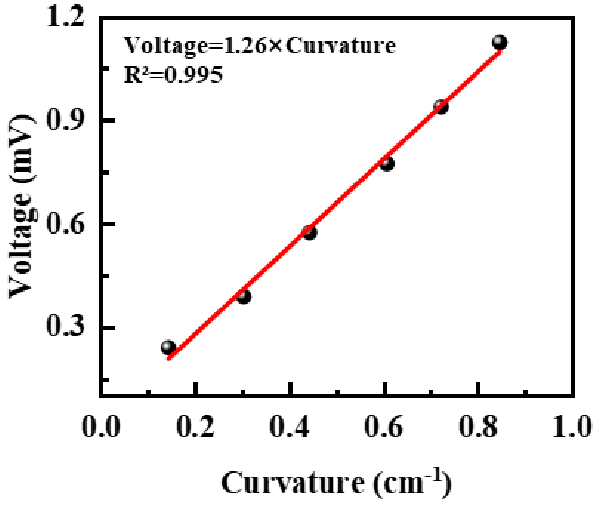

Figure S21. Output voltage as a function of bending curvature of the PPy@G-BC/BOPP lihgt-driven actuator.

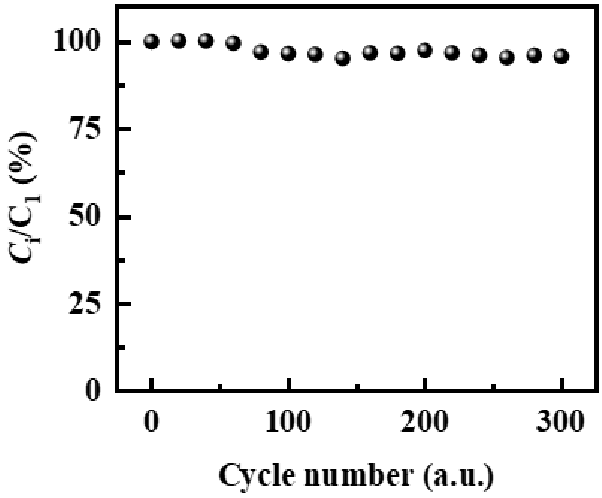

Figure S22. Repeatability test of the bending curvature of PPy@G-BC/BOPP lihgt-driven actuator with NIR light irradiation for 300 cycles (light power density of 200 mW cm^{-2}). C_1 represents the maximum curvature of the actuator under the first NIR light irradiation, while C_i represents the maximum curvature of the actuator at the i-th cycle.

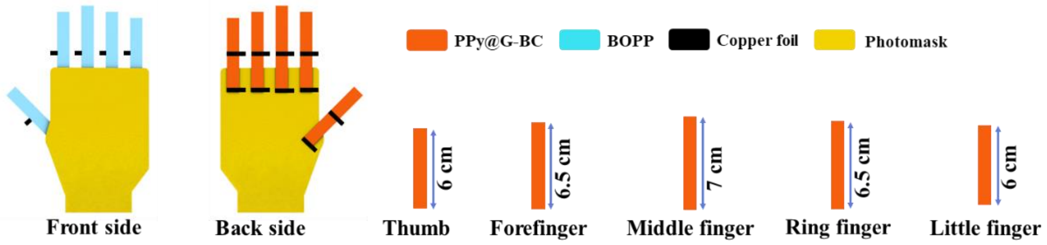

Figure S23. Dimensions of the light-driven bionic hand.

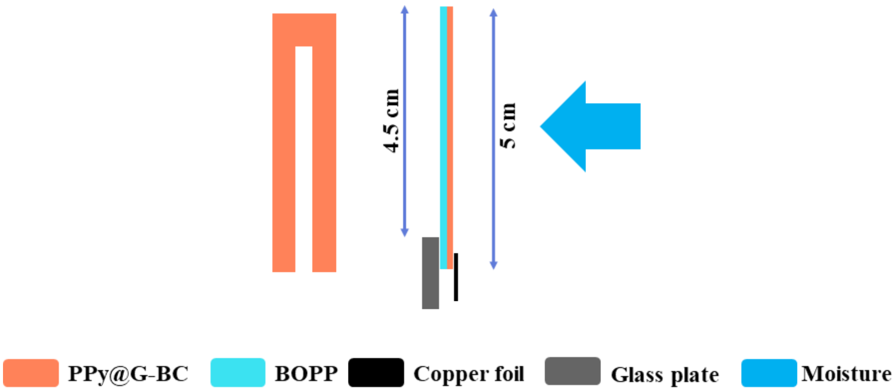

Figure S24. Dimensions of the PPy@G-BC/BOPP actuator when humidified.

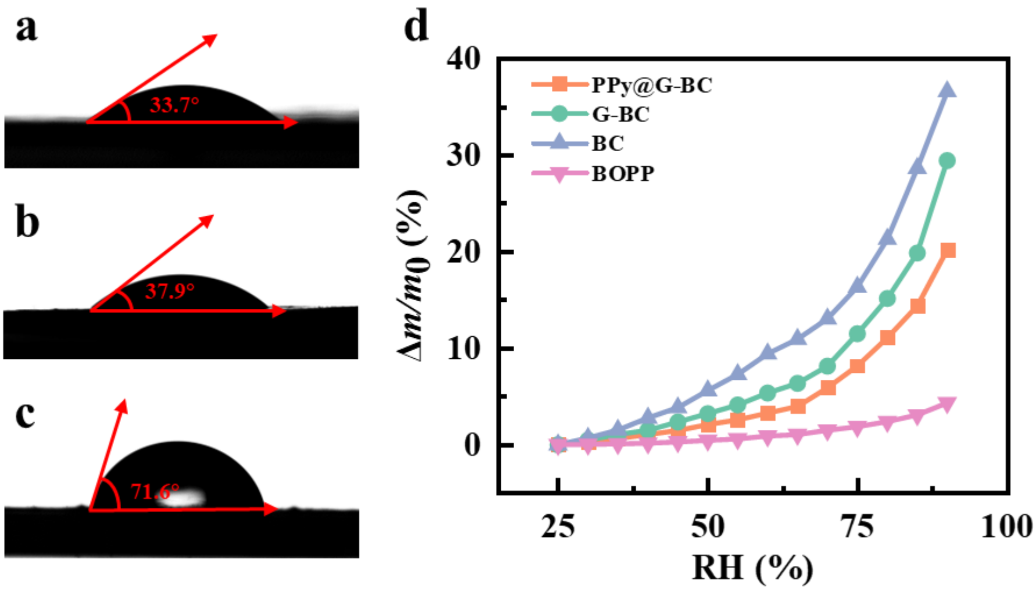

Figure S25. Hydrophilicity test. (a) Water contact angle of the G-BC film. (b) Water contact angle of the PPy@G-BC film. (c) Water contact angle of the BOPP film. (d) Water absorption of BC film, G-BC film, BOPP film and PPy@G-BC film.

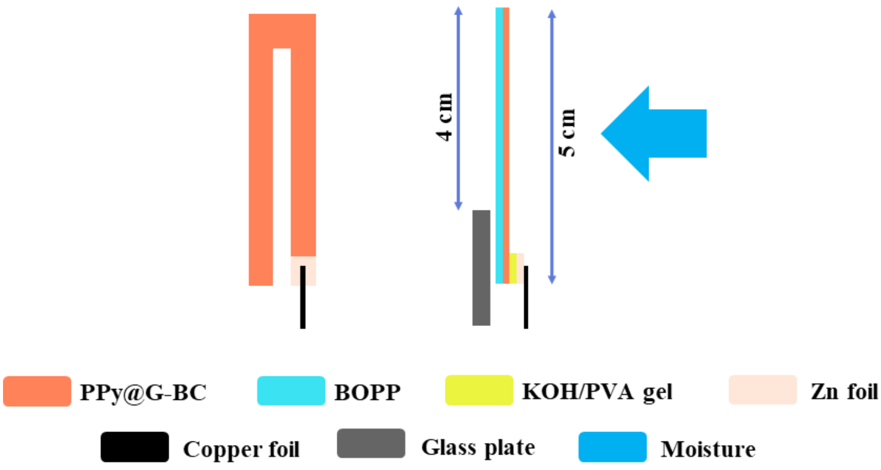

Figure S26. Dimensions of the PPy@G-BC/BOPP actuator integrated with zinc-air battery when humidified.’

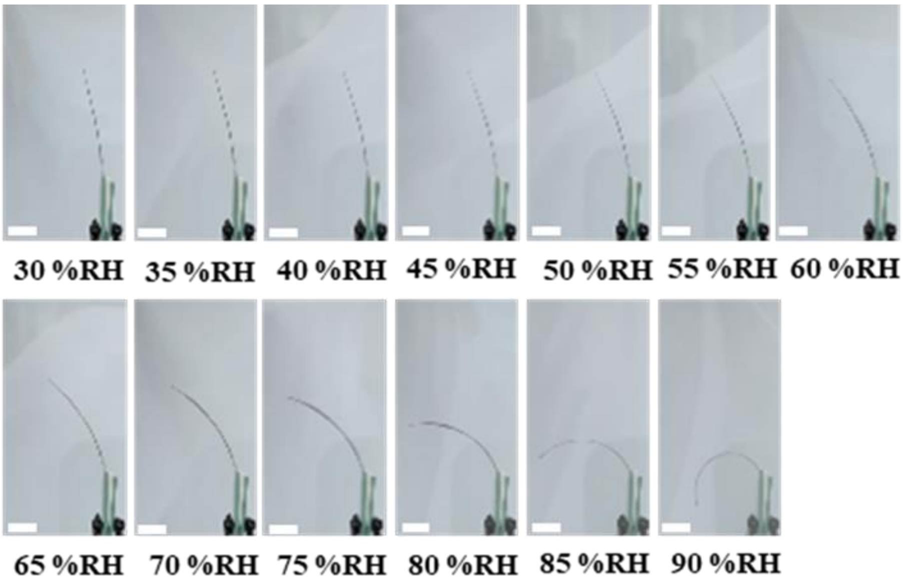

Figure S27. Optical photos of the PPy@G-BC/BOPP actuator integrated with zinc-air battery when humidified. Scale bar: 1 cm.

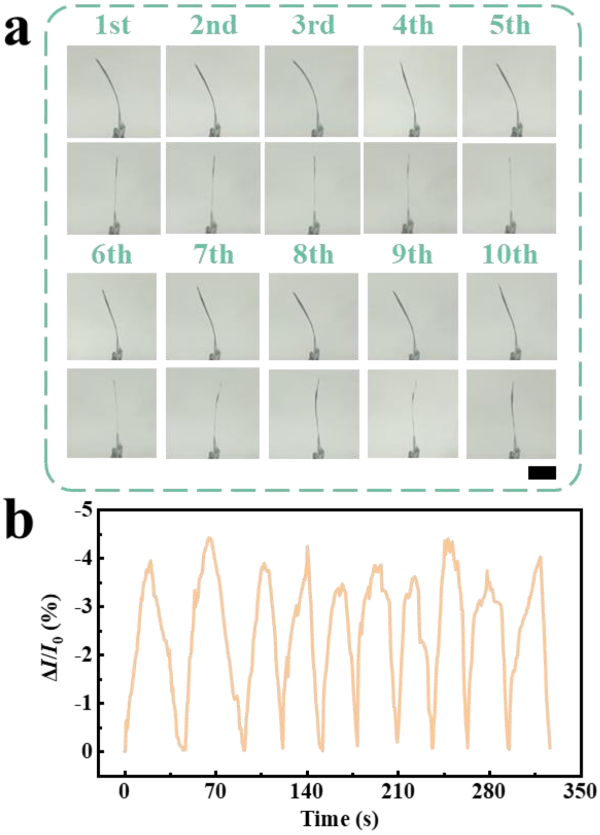

Figure S28. (a) Optical photos of the deformation of the PPy@G-BC/BOPP actuator under cyclic light/humidity dual stimulation. Scale bar: 2 cm. (b) Durable performance of the relative current change rate of PPy@G-BC/BOPP actuator under cyclic light/humidity dual stimulation.

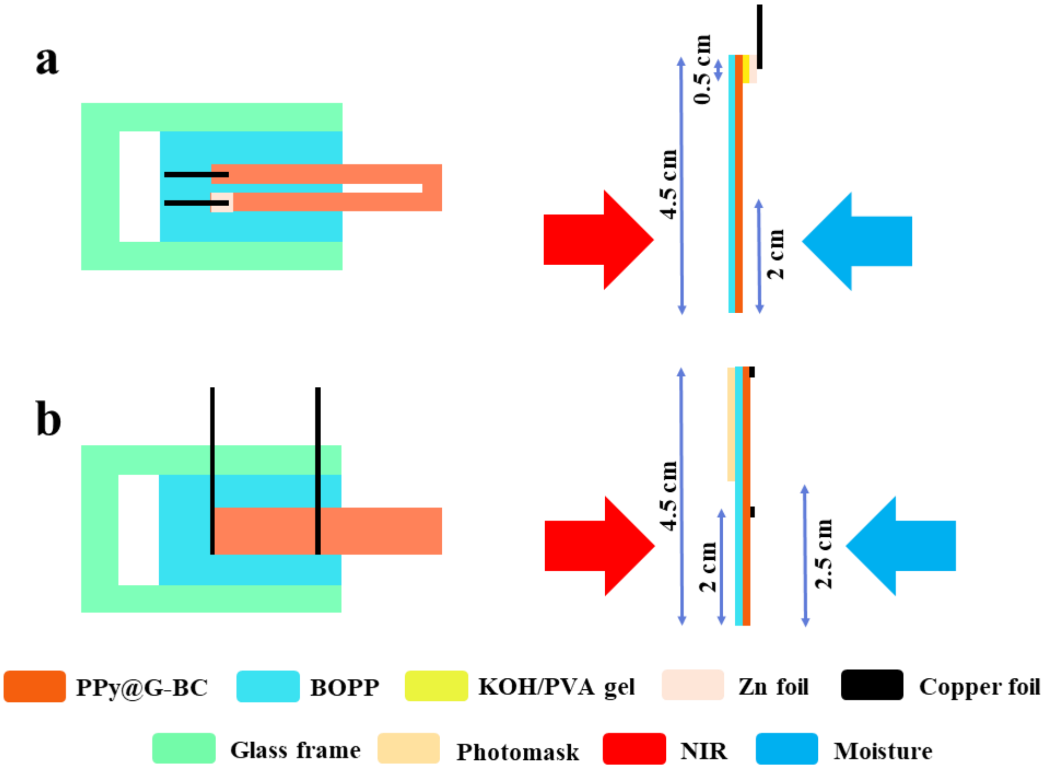

Figure S29. Dimensions of the intelligent gripper with multi-responsive actuation and self-powered multi-mode sensing. (a) The PPy@G-BC/BOPP actuator with a self-powered humidity sensing function. (b) The PPy@G-BC/BOPP actuator with a self-powered temperature sensing function.

Note S1. Bending curvature calculation principle for PPy@G-BC/BOPP actuator

The parameters are defined as follows (shown in Figure S18):

L: The length of the PPy@G-BC/BOPP actuator.

ρ: The radius of the arc of the curved PPy@G-BC/BOPP actuator.

x: The horizontal free-end displacement of the PPy@G-BC/BOPP actuator.

y: The vertical free-end displacement of the PPy@G-BC/BOPP actuator.

$\theta/2$: The chord tangent angle of the PPy@G-BC/BOPP actuator.

θ: The bending angle of the arc of the PPy@G-BC/BOPP actuator.

The curvature is defined as the reciprocal radius ($1/\rho$).

The chord tangent angle is given by

$$\frac{\theta}{2} = arctan\frac{x}{y}$$

As the bending angle is given by

$$\theta = \frac{L}{\rho}$$

the curvature $1/\rho$ is deduced as

$$k = \frac{1}{\rho} = \frac{\theta}{L}$$

Hence, the curvature of the actuator can be calculated by achieving the bending angle and length of the actuator.

Note S2. Overall discharge reactions of flexible zinc-air battery

At the air cathode, oxygen (O_2) absorbs electrons from the air and combines with protons (H^+) in water (H_2O) to form hydroxide ions (OH^-). The reaction of the air cathode is:

$O_2 + 4e^- + 2H_2O \rightarrow 4OH^-$

At the metal anode, zinc metal loses two electrons and gets oxidized to Zn^{2+}, which accumulates at the anode. The reaction of the zinc anode is:

$Zn - 2e^- \rightarrow Zn^{2+}$ $Zn^{2+} + 2OH^- \rightarrow Zn(OH)_2 \rightarrow ZnO + H_2O$

The electrons generated by this reaction flow through the external circuit to the cathode, thus completing the closed circuit of the zinc-air battery. At the same time, zinc ions (Zn^{2+}) and hydroxide ions (OH^-) move through the electrolyte to maintain the charge balance in the battery. The overall reaction is:

$2Zn + O_2 \rightarrow 2ZnO$

Note S3. Calculation of thermoelectric figure of merit

The thermoelectric figure of merit (*ZT*) is a quality factor that characterizes the comprehensive thermoelectric performance of a material (Mater. Today Phys., 2018, 4, 50):

$$ZT = \frac{S^2\sigma}{\kappa}T = \frac{PF}{\kappa}T$$

where *S*, σ, *T*, κ, *and PF* are the Seebeck coefficient, electrical conductivity, absolute temperature, thermal conductivity, and power factor, respectively. The higher the *ZT* value, the better the comprehensive thermoelectric properties of the material, and the higher the energy conversion efficiency of the corresponding thermoelectric device.

Table S1. Comparison of mechanical properties of PPy@G-BC film with similar flexible materials in the field of multifunctional actuator.

Materials	Tensile stress	Tensile strain	Young's module	Ref.
PANI@CNT-BC film	16.5 MPa	~ 0.6%	3.63GPa	**Chem. Eng. J., 2022, 452, 139146**
PIQA/CNS film	56.1 MPa	4.6%	~ 2.3 GPa	**Adv. Mater., 2021, 33, 2104558**
$Ti_3C_2T_x$/Bamboo nanofiber	11.3 MPa	~ 0.6%	1.5 GPa	**Nanoscale, 2023, 15, 18842-18857**
MXene film	44.5 MPa	1.4%	4.8 GPa	**Nano Energy, 2022, 103, 107848**
IPTS-MXene film	~ 140 MPa	2.2 %	~ 8 GPa	**ACS Nano, 2021, 15, 5294−5306**
PVDF–CB/PEA/PAM film	16 MPa	47%	~ 0.33 GPa	**J. Mater. Chem. A, 2022,10, 25337-25346**
Sodium alginate film	~ 50 MPa	~ 5%	/	**Adv. Funct. Mater., 2023, 2311398**
PVA/PAAm/TA film	75 MPa	~ 4%	7.1 GPa	**Adv. Mater. Technol. 2023, 8, 2300603**
CNTS film	~ 80 MPa	3.25 %	~ 3.4 GPa	**Nano Letters, 2023, 23, 6504−6511**
CNF/PVA/LM film	~ 40 MPa	~ 55%	~ 0.8 GPa	**ACS Nano, 2023, 17, 24042−24054**
**PPy@G-BC film**	**93.53 MPa**	**3.08%**	**5.55 GPa**	**Our work**

Table S2. Comparison of thermoelectric properties of PPy@G-BC film with PPy or graphene based thermoelectric materials.

Materials	*S* (μV K^{-1})	*σ* (S cm^{-1})	*κ* (W m^{-1} k^{-1})	*PF* (μW m^{-1} K^{-2})	*ZT*	Ref.
Graphene/ZnO/cement	1.41×10^5	17.9	0.92	28	~0.01	**Energy, 2020, 198, 117396**
PPy/SWCNT	~20	1.067×10^4	0.516	365.2	0.203	**Chem. Eng. J., 2022, 443, 136536**
PPy/PANI/CB	38.45	0.1698	0.215	0.0251	4.307×10^{-5}	**J. Colloid Interface Sci., 2023, 630, 46-60**
PPy/FrGO-WO_3	8	10.87	0.127	0.0696	1.63×10^{-4}	**Synthetic Met., 2023, 298, 117427**
SWCNT/PPy	47	1736.37	0.12	382	0.11	**Nanomaterials, 2022, 12, 2582**
SnSe/graphene	572	~25	0.18	~250	1.06	**Nanoscale, 2020, 12, 12760-12766**
PDG/PANI	604	14.5	0.22	/	0.74	**ACS Appl. Mater. Interfaces, 2018, 10(5), 4946-4952**
PPy/graphene	31.74	~100	1.09	10.24	2.80×10^{-3}	**Rsc Adv., 2014, 4, 46187-46193**
PPy-MWCNT-$(COOH)_3$-NiO	29	69.23	~0.116	5.28	1.51×10^{-2}	**ChemNanoMat, 2023, e202300486**
GO-MWCNT	70	~0.57	0.056	/	~0.001	**Compos. Part B-Eng., 2015, 83, 317-322**
**PPy@G-BC (*T*=298.15 K)**	**42.7**	**41.67**	**0.109**	**7.61**	$\mathbf{20.82\times10^{-3}}$	**This work**
